# Effects of silybin supplementation on growth performance, serum indexes and liver transcriptome of Peking ducks

**DOI:** 10.3389/fvets.2023.1325115

**Published:** 2024-01-03

**Authors:** Ziyue Zhang, Bozhi Shi, Xueze Lv, Yingchao Dong, Lei Li, Zhaofei Xia

**Affiliations:** ^1^College of Veterinary Medicine, China Agricultural University, Beijing, China; ^2^Beijing General Animal Husbandry Station, Beijing, China; ^3^Feed Research Institute, Chinese Academy of Agricultural Sciences, Beijing, China; ^4^College of Veterinary Medicine, Yunnan Agricultural University, Kunming, China

**Keywords:** silybin, growth performance, immunity levels, liver, transcriptome

## Abstract

As an emerging feed additive extracted from the traditional herb milk thistle, silybin has few applications and studies in Peking ducks. The aim of this study was to explore the practical significance of silymarin application in Peking ducks and to provide more theoretical support for the application of silymarin in livestock and poultry production. A total of 156 1-day-old healthy Peking ducks were randomly divided into four groups and supplemented with 0 mg/kg (control group), 400 mg/kg (S400), 800 mg/kg (S800) and 1,600 mg/kg (S1600) of silybin in the diets at day 14, to investigate the effects of silymarin on the growth, serum indexes and liver transcriptome of Peking ducks. The whole experiment lasted until day 42, and the sample collection was scheduled to take place in the morning. A substantial inprovement in average daily gain (ADG) and a decrease in feed conversion ratio (FCR) occurred in the S1600 group on days 14–28 compared to the control group (*p* < 0.05). The FCRs of other additive groups in the same period showed the same results. Supplementation of diets with silybin significantly increased serum IgA levels and when 1,600 mg/kg of silybin was given, levels of TNF-α and IL-6 were also significantly decreased (*p* < 0.05). In addition, we observed that the S1600 group had a significantly lower (*p* < 0.05) glutamine transaminase and an increased (*p* < 0.05) T-SOD level in the S400 group (*p* < 0.05). Liver transcriptome sequencing showed that 71 and 258 differentially expressed genes (DEGs) were identified in the S400 and S1600 groups, respectively, compared with the control group. DEGs related to cell composition and function, antigen processing and presentation were up-regulated, while DEGs related to insulin resistance and JAK–STAT were down-regulated. Conclusively, silybin can be used as a feed additive to improve the growth performance and health status of Peking ducks.

## 1 Introduction

With a history of more than 400 years, Peking ducks are not only an important part of China’s livestock and poultry breeding industry, but also a world-famous meat duck breed, which occupies a pivotal position in both domestic and international duck breeding industries. With the passage of time, the waterfowl production industry continues to develop and progress, and the global duck meat production is increasing year by year (1). Asia is the largest meat duck producing region in the world, and China is the main meat duck-producing country (2). That means there is a higher demand on the productivity of broiler ducks, as well as higher demands on nutritional conditions, feeding management and other factors (3). Although the use of growth-promoting antibiotics can significantly improve the growth performance of birds and the corresponding efficiency, considering the subsequent adverse effects caused by the use of antibiotics, many countries and regions have proposed and implemented measures to reduce the use of antibiotics in recent years (4, 5). The importance of finding and researching the use of green and safe alternatives is particularly urgent. Traditional Chinese medicines (TCM) and their extracts are getting more and more attention due to their excellent performance in food, medicine, farming, and amelioration of pathogen infections.

Silybin is a flavonoid extracted from the herb *Silybum marianum* of the Asteraceae family, which is effective in the treatment of Hepatitis, Cirrhosis, Viral Liver Disease, Alcoholic and Non-Alcoholic liver disease, and other liver diseases, which can play a role in antioxidant by scavenging free radicals and inhibiting lipid peroxidation (6–12). Previous studies have found that silybin reduces the formation of free radicals in injured cells, which could also serve as an explanation for the hepatoprotective properties of silybin (13). Furthermore, silybin has a protective effect on the liver of ducks fed diets containing mycotoxin (14). Some scholars have found that silybin activates the body’s antioxidant system by increasing sulfhydryl concentration and reducing power (15). Studies on silybin in different animal bodies have gradually increased in recent years, but there are fewer relevant studies on the application of silybin in Peking ducks. Having considered the different reactions that may occur in different animal organisms after the introduction of silybin into the body, we believed that studies related to the effects of silybin on Peking ducks were necessary.

The liver is not only an important immune organ of the body, but also an important hub of many physiological processes (16). It has a high capacity for repair and regeneration and can usually respond quickly to external stimuli (17). The transcriptome is an important tool for studying the dynamics of gene expression, analyzing and identifying disease markers, and facilitating the discovery of new targets (18). RNA sequencing (RNA-Seq) is a common method of genomic analysis with high resolution, high throughput, and high sensitivity, through which the technology can reveal more new transcribed regions or capture the transcriptome more accurately in different environments or over time (19, 20). Currently, no relevant studies have been found on the effect of feeding diets containing silybin on gene expression in the liver of Peking ducks. Therefore, the application of transcriptomics to study the expression of hepatic genes in Peking ducks fed diets containing silybin is beneficial to further understanding the effects of silybin on the growth and development of Peking ducks, and may also provide further information on the application of silybin in animal diets.

In order to clarify the effects of dietary silybin supplementation on Peking ducks, we set up different silybin supplementation levels to carry out relevant experiments, then investigated the growth performance, serum biochemical levels, antioxidant levels, immune factor levels, inflammatory factor levels, and hepatic transcript levels of Peking ducks.

## 2 Materials and methods

### 2.1 Animal ethics and reagents

All procedures were carried out in accordance with the provisions of the Animal Welfare and Animal Experimental Ethics Review Committee of China Agricultural University. The animal ethics number is AW61303202-2-3. Animal experiments were conducted in Nankou Pilot Test Base of the Chinese Academy of Agricultural Sciences. Silybin was purchased from Shaanxi Haokang Biotechnology Co., LTD., with a concentration of 98%.

### 2.2 Animals, diets, and experimental design

One hundred and fifty-six 1-day-old healthy Jing Dian Peking ducks were randomly divided into four treatment groups with three replicates of 13 ducks each. 0.400 mg/kg, 800 mg/kg, and 1,600 mg/kg silybin were added to the diets of the four treatment groups from 14 days of age. The basic diet was formulated in accordance with NY/T 2122–2012 “Feeding Standards for Meat Ducks,” and the composition and nutritional levels of the basic diet were shown in Tables 1, 2. The weight of all ducks was recorded at 14 days of age, and again on the 28th and 42nd day of the experiment, then the average daily gain (ADG), average daily feed intake (ADFI) and feed conversion ratio (FCR) were calculated. Before the experiment begins, the duck house should be cleaned and disinfected thoroughly. Clean the manure at 7 a.m. every day, and clean the sink and trough once a week. During the experiment, ducks were able to feed and drink freely, and performed immune procedures normally.

**Table 1:** Composition of basal diets (air-dry basis, %).

Items	Day 1–14	Day 15–42
Corn	56.00	60.24
Soybean meal	32.69	24.67
Wheat midding	5.00	9.00
Soybean oil	2.10	1.80
Phytases	0.02	0.02
CaHPO_4_	1.00	1.60
Limestone	1.50	1.20
DL-Met	0.15	0.12
L-Lys	0.20	0.10
Vitamin premix^a^	0.02	0.02
Trace mineral premix^b^	0.20	0.20
NaCl	0.35	0.30
Cholie chloride (50%)	0.24	0.20
Ethoxyquin (33%)	0.03	0.03
Maifanite	0.50	0.50
Total	100.00	100.00

^a^The vitamin premix provided the following per kilogram of diet: vitamin A, 12,500 IU; vitamin D3, 3,500 IU; vitamin E, 20 IU; vitamin K3, 2.65 mg; thiamin, 2.00 mg; riboflavin, 6.00 mg; pyridoxin, 3.00 mg; VB12, 0.025 mg; biotin, 0.0325 mg; folic acid, 12.00 mg; pantothenic acid, 50 mg; nicotinic acid, 50.00 mg. ^b^The mineral premix provided the following per kilogram of diet: Cu, 6 mg; Fe, 80 mg; Zn, 40 mg; Mn, 100 mg; Se, 0.15 mg; I, 0.35 mg.

**Table 2:** Nutrient levels of basal diets (calculated value).

Items	Day 1–14	Day 15–42
ME	12.31	12.53
CP	19.52	16.83
Lys	1.12	0.87
Met	0.46	0.39
Ca	0.88	0.89
AP	0.29	0.39
TP	0.54	0.62
Met+Cys	0.79	0.69

ME, Metabolizable energy; CP, Crude protein; Lys, Lysine; Met, Methionine; Ca, Crude ash; AP, Available phosphorus; TP, Total phosphorus; Cys, Cysteine.

### 2.3 Sample collection

After fasting for 12 h in advance, three Peking ducks were randomly taken from each replicate group at 7:00 am on day 28 and day 42 of the experiment, respectively, and were labeled and weighed. 10 mL of blood was collected from the jugular vein and centrifuged at 4000 r for 10 min at 4°C, and the serum was stored at −20°C for further testing. The liver tissues of mung bean grain size were taken in 2 mL RNA-Free enzyme lyophilization tubes and stored in liquid nitrogen first, and then transferred and stored at −80°C.

### 2.4 Analysis of serum component

Serum ALP, ALT, ALB, TP and γ-GT levels were measured by an automatic biochemical analyzer. The levels of serum IgA, IgG and IgM, inflammatory factors IL-1β, IL-4, IL-6, IL-10 and TNF-α were determined using an enzyme marker; the levels of antioxidant indicators T-SOD, T-AOC, CAT and GSH-PX were determined using a spectrophotometer. The assay kits used for all the above indicators were provided by Beijing Kangjia Hongyuan Biotechnology Co. All of the above indicators were measured in accordance with the instructions.

### 2.5 Total RNA extraction and sequencing

The total RNA in liver tissue was extracted by trizol, and the Illumina Stranded mRNA Library Prep Kit was used to construct a library with 1 μg total RNA of each sample. The mRNA isolated from total RNA by Oligo dT should first be randomly interrupted with buffer under suitable conditions, and a strand of cDNA should be synthesized using the mRNA as a template under the action of reverse transcriptase using random primers. Subsequently, the synthesis of the second chain was carried out. The uneven end of the synthesized cDNA is repaired and an “A” base is added to the 3′ end. The above products were purified and sorted. PCR amplification was performed with the sorted products, and after quantification with Qubit 4.0, sequencing was performed using the Illumina NovaSeq 6000 platform.

### 2.6 Data processing and analysis

The raw sequencing data was filtered using fastp software to obtain high quality sequencing data. The raw data after quality control was then compared to the reference genome using HiSat2, and the results were also compared for quality assessment. Then the expression levels of genes and transcripts were quantitatively analyzed by RSEM.^1^ In order to identify differentially expressed genes among different samples and study their functions, the data were screened and analyzed using the software DESeq2. A gene was considered to be differentially expressed gene (DEG) when it satisfied both FDR < 0.05 & |log2FC| ≥ 1. Subsequent analysis of the data was performed using the Gene Ontology (GO)-based Functional Enrichment and Annotation tool and the Kyoto Encyclopedia of Genes and Genomes (KEGG).

### 2.7 Statistical analysis

Excel 2021 was used for enter and initially organization of experimental data, then the data were subjected to one-way ANOVA analysis using SPSS 26.0. When the *p*-value below 0.05 were considered to be significantly different. Images were processed by GraphPad Prism 9.0.

## 3 Results

### 3.1 Growth performance

**Figure 1:**
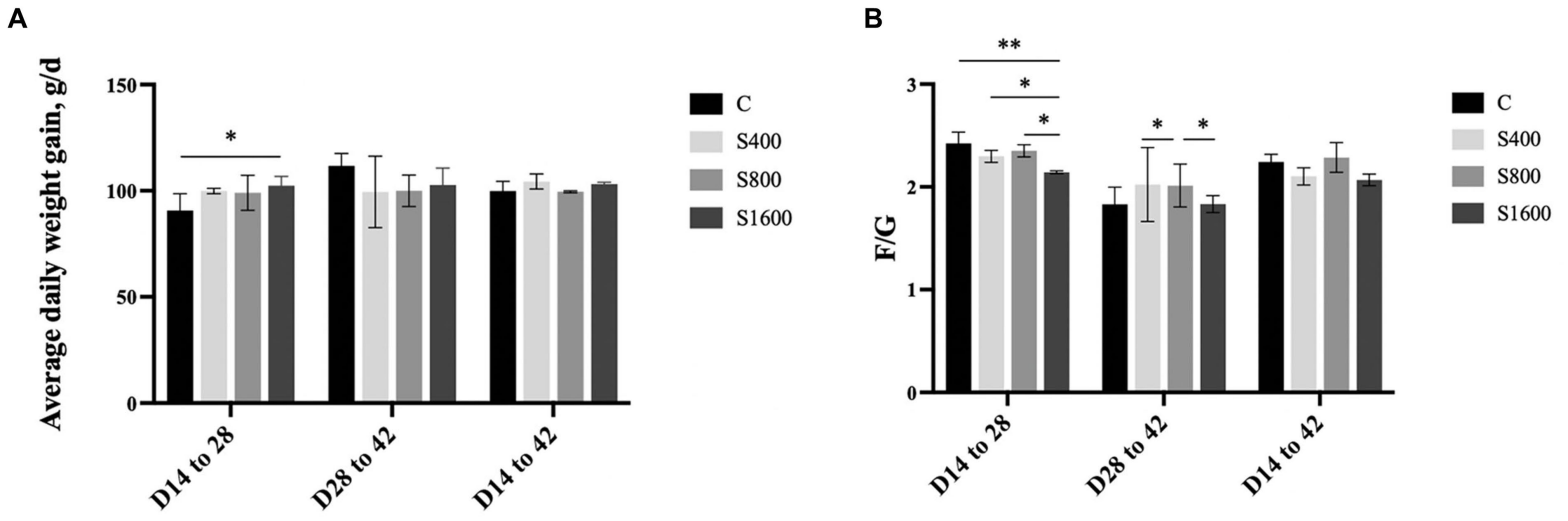
Effect of silybin on average daily gain and feed conversion rate. **(A)** Average daily weight gain in different stages. **(B)** Feed conversion rate in different stages. *p < 0.05; **p < 0.01.

The effects of dietary supplementation with silybin on the growth performance of Peking ducks were shown in the result (Table 3 and Figure 1). From day 14 to day 28, ADG and FCR of Peking ducks in the S1600 group were significantly increased (*p* < 0.05); however, there were no significant differences in the ADG, ADFI and FCR among the treatment groups from day 28 to day 42. From day 14 to day 42, there was a decreasing trend in ADFI and FCR in S400 and S1600 groups compared with the control group, but the differences were not significant.

**Table 3:** Growth performance during the growing season of adding silybin.

Items	Groups				*p* value
	C	S400	S800	S1600	
*Day 14–28*					
Average daily gain, g/d (ADG)	90.698^a^	99.896^ab^	99.023^ab^	102.430^b^	0.185
Average daily feed intake, g/d (ADFI)	219.381	229.469	233.022	219.505	0.616
Feed conversion rate (FCR)	2.422^a^	2.297^a^	2.351^a^	2.142^b^	0.007
*Day 28–42*					
Average daily gain, g/d (ADG)	111.746	99.444	100.000	102.698	0.483
Average daily feed intake, g/d (ADFI)	204.190	197.106	200.275	188.150	0.458
Feed conversion rate (FCR)	1.831	2.022	2.013	1.832	0.595
Day 14–42					
Average daily gain, g/d (ADG)	99.833	104.353	99.512	103.123	0.194
Average daily feed intake, g/d (ADFI)	223.745	219.288	227.521	213.276	0.335
Feed conversion rate (FCR)	2.243^abc^	2.102^a^	2.286^b^	2.068^ac^	0.062

In the same row, values with different superscript indicate a significant difference (*p* < 0.05).

### 3.2 Serum indicators

**Figure 2:**
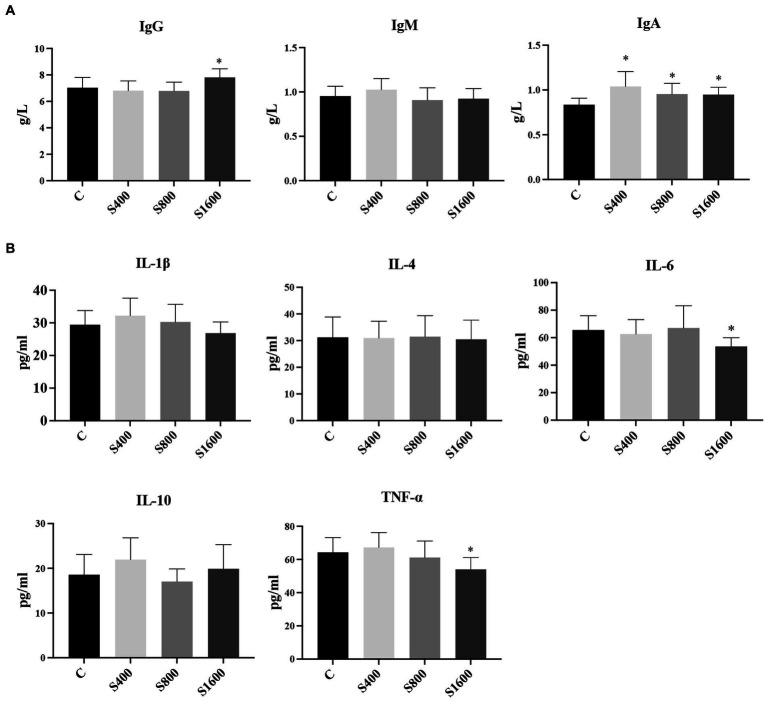
Serum immune levels and inflammatory indices affected by silybin. **(A)** Effect of silybin on IgG, IgA and IgM. **(B)** Effect of silybin on the expression of inflammatory factors. *p < 0.05; **p < 0.01.

**Figure 3:**
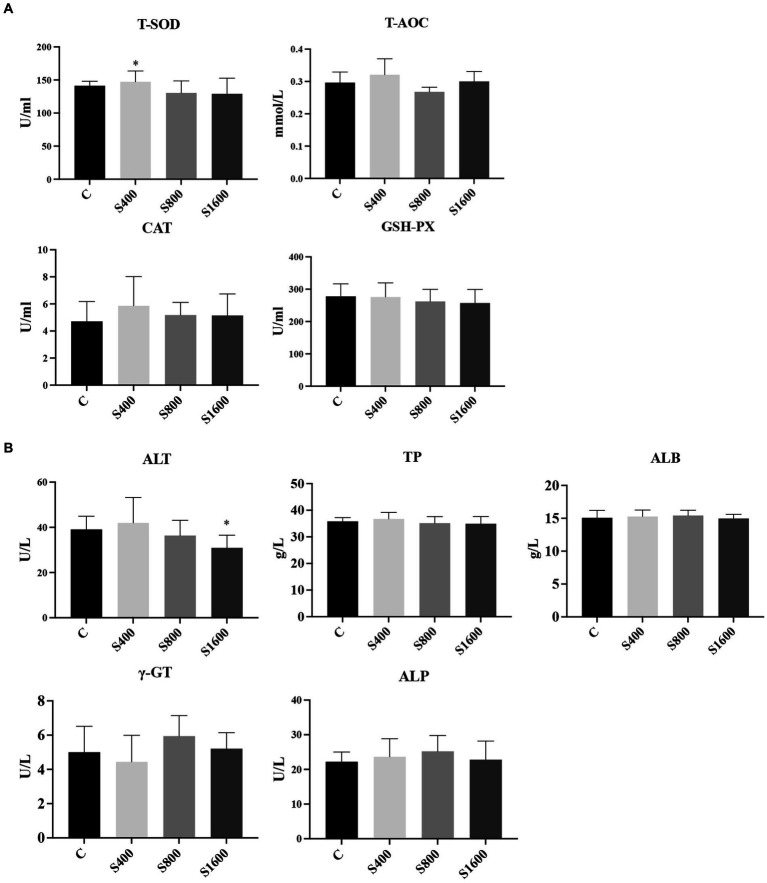
Serum biochemical and antioxidant as influenced by silybin. **(A)** Effect of silybin on serum antioxidant levels. **(B)** Effects of silybin on biochemical levels. *p < 0.05; **p < 0.01.

The results of the serum index tests were shown in the figures (Figures 2, 3). At day 42, IgA content in the silybin-added group were all significantly elevated compared with the control group (*p* < 0.05); IgG in S1600 group was also elevated, with significant differences (*p* < 0.05) (Figure 2A). The levels of IL-6 and TNF-α in S1600 group were both decreased significantly (*p* < 0.05) and IL-10 was increased. But there was no effect on the levels of IL-1β and IL-4 at day 42, while IL-10 in S400 group was increased at day 42 with a non-significant difference (Figure 2B). Compared with the control group, the content of T-SOD in S400 group was significantly increased (*p* < 0.05), while T-AOC and CAT were also increased, but there was no significant difference in the content of GSH-PX in the additive group compared with the control group (Figure 3A). ALT was observed to be significantly different between groups S1600 and control (*p* < 0.05), and there was a tendency for a decrease in γ-GT in S400 group (Figure 3B).

### 3.3 RNA sequencing data statistics

A total of nine samples were completed for transcriptome analysis, and 66.1 Gb Clean Data was obtained, with Clean Data of each sample reaching more than 6.04 Gb, and the percentage of Q30 bases was above 93.63%. The details of these sequencing data are reflected in Table 4.

**Table 4:** RNA sequencing data statistics.

Sample	Raw reads	Raw bases	Clean reads	Clean bases	Q20 (%)	Q30 (%)
QK5	55,234,502	8,340,409,802	54,751,870	8,115,627,975	98.12	94.55
QK6	63,316,286	9,560,759,186	62,681,984	9,300,686,701	98	94.24
QK5_6	45,726,180	6,904,653,180	45,312,194	6,741,760,126	97.98	94.17
QK10	53,815,644	8,126,162,244	53,379,050	7,951,670,230	98.01	94.23
QK12	45,793,940	6,914,884,940	45,352,738	6,731,750,720	98.05	94.37
QK10_12	51,914,606	7,839,105,506	51,372,750	7,633,608,369	97.91	94.04
QK13	50,235,290	7,585,528,790	49,724,084	7,385,028,335	97.75	93.63
QK14	41,112,750	6,208,025,250	40,708,590	6,039,480,909	97.94	94.12
QK13_14	42,377,138	6,398,947,838	41,949,394	6,196,310,790	98.13	94.59

### 3.4 Statistics of differentially expressed genes (DEGs)

**Figure 4:**
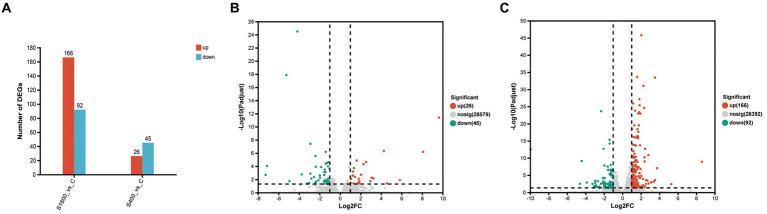
Up-regulation and down-regulation of differentially expressed genes. **(A)** The expression of differentially expressed genes between S1600 and C group, and S400 and C group. **(B)** Volcano plot of differentially expressed genes between S400 and C group. **(C)** Volcano plot of differentially expressed genes between S1600 and C group.

**Figure 5:**
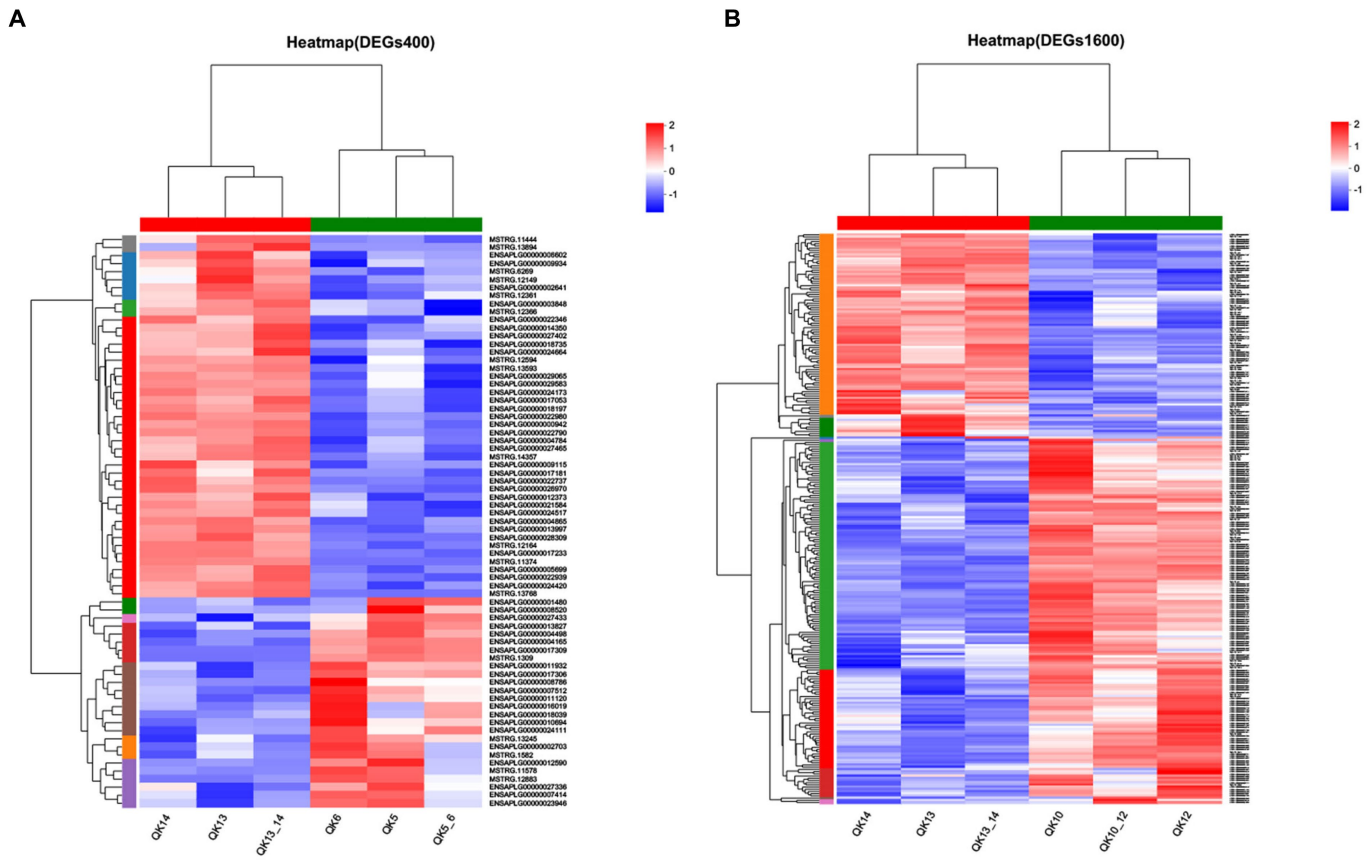
Heatmap of gene expression between silybin-added and control groups. **(A)** Heatmap of gene expression in S400 and C groups. **(B)** Heatmap of gene expression in S1600 and C groups.

The differentially expressed genes between different treatment groups and control group were analyzed, respectively. The results showed that a total of 258 differentially expressed genes were identified between the S1600 group and the control group, of which 166 DEGs were up-regulated and 92 DEGs were down-regulated; 71 DEGs were identified between the S400 group and the control group, of which 26 were up-regulated and 45 were down-regulated (Table 5; Figure 4). Gene clustering analysis of the expression models of DEGs in the two grouping scenarios showed that the gene expression patterns were similar within each grouping scenario, suggesting good reproducibility of the samples between the groups and a high degree of gene set reliability (Figure 5). Details of the expression of differentially expressed genes are shown in Supplementary Tables S1, S2.

**Table 5:** Differentially expressed gene statistics.

DIFF group	Total DEGs	Up	Down
S1600 vs. C	258	166	92
S400 vs. C	71	26	45

### 3.5 Differentially expressed genes (DEGs) annotation and enrichment analysis

**Figure 6:**
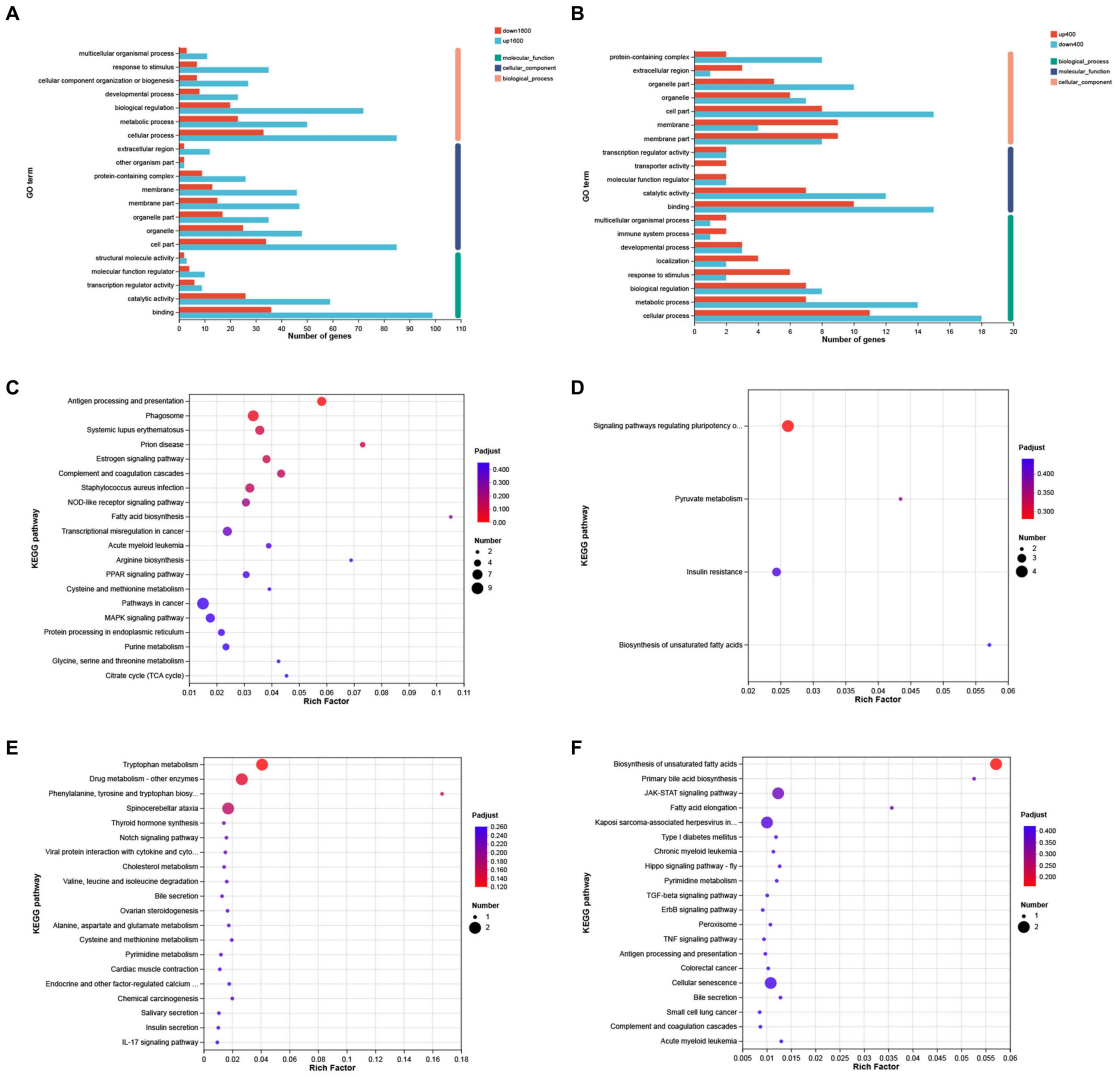
GO annotation and KEGG enrichment analysis of differentially expressed genes. **(A)** GO annotation analysis of differentially expressed genes in S1600 and C groups. **(B)** GO annotation analysis of differentially expressed genes in S400 and C groups. **(C)** KEGG enrichment analysis of differentially expressed genes up-regulated in S1600 and C groups. **(D)** KEGG enrichment analysis of differentially expressed genes down-regulated in S1600 and C groups. **(E)** KEGG enrichment analysis of differentially expressed genes up-regulated in S400 and C groups. **(F)** KEGG enrichment analysis of differentially expressed genes down-regulated in S400 and C groups. The vertical axis represents different GO terms **(A,B)** or pathways **(C–F)**, and the horizontal axis represents rich factor.

The results of GO analysis showed that 258 DEGs between S1600 group and control group were annotated to the three major categories of biological processes, cellular components, and molecular functions, with the largest number of DEGs being annotated to the biological process term (Figure 6A). The most abundant GO Terms in biological processes is cellular processes (GO: 0009987), with 118 DEGs annotated under this item; the cellular component (GO: 0044464) and organelle (GO: 0043226) entries in the cellular component were annotated to the most DEGs, with 119 and 72 annotations, respectively. Binding (GO: 0005488) function is the most abundant GO Terms in molecular function. KEGG enrichment analysis of 258 differentially expressed genes showed that up-regulated DEGs were significantly enriched in antigen processing and presentation, phagosome, and complement pathways (Figure 6C); while down-regulated genes were mainly enriched in pyruvate metabolism, insulin resistance, and polyunsaturated fatty acid synthesis in stem cells (Figure 6D). GO analysis showed that cellular fractions were annotated to the maximum of the number of differentially expressed genes in the S400 and control groups. The entries with the highest number of differentially expressed genes annotated to the three broad categories were consistent with the former (Figure 6B). These two groups of up-regulated differentially expressed genes were significantly enriched in amino acid metabolism and synthesis, and drug metabolism pathways (Figure 6E); whereas down-regulated differentially expressed genes were enriched in unsaturated fatty acid synthesis pathways and JAK–STAT signaling pathway (Figure 6F). Specific information on the results of KEGG enrichment analysis of differentially expressed genes is shown in the Supplementary Tables S3, S4.

## 4 Discussion

Due to the irrational use of antibiotics in livestock and poultry farming, drug residues and the emergence of drug-resistant bacteria have seriously jeopardized human health (21, 22). Currently, one of the main measures to deal with this situation is to reduce and gradually abandon the use of some antibiotics, thus finding suitable and effective alternatives has become a major trend today (23). It has been proved that the application of herbs and related substances such as Chinese herbs, Chinese herbal extracts, Chinese herbal ferments and other related substances in livestock and poultry production can play a role in lowering the FCR, improving the meat quality, enhancing the intestinal barrier function, promoting the metabolism of proteins and other effects, then lowering the cost of feeding (24–26). Silybin is extracted from the traditional plant milk thistle, which has outstanding anti-inflammatory and antioxidant effects. Among the many extracts, silybin has important biological effects and can be used in almost all common liver diseases (27). This study aims to investigate the effects of dietary silybin on the growth performance and liver transcriptome of Peking ducks, and to provide a theoretical basis for the application of silybin in feed additives. The results showed that dietary silybin supplementation can significantly reduce the FCR of Peking ducks aged 14–28 days, and the addition of 1,600 mg/kg also significantly increased the average daily gain of Peking ducks at the same period, similar findings were found in Shanmugam’s study (28), where they observed that dietary supplementation of milk thistle extract can increase body weight and improve growth performance of broilers. The research results of Tedesco also confirmed that plant extracts such as magnolol and Astragalus polysaccharide can significantly improve the growth performance, enhance meat quality and reduce the FCR in poultry animals such as chickens and ducks (29–31). Therefore, we believe that silybin has a positive regulatory effect on improving the growth performance of animals. Interestingly, however, in our study, silybin had no significant effect on the growth performance of Peking ducks at day 28–42. Similarly, it was found that the addition of *Cornus officinalis* extract did not significantly affect its FCR and growth performance in the application of broilers (32). There is a strong correlation between the growth performance of birds and the development of internal organs, in which the development of the intestine plays an important role, and the digestion and absorption of nutrients mainly occur in the small intestine. Therefore, we hypothesized that the regulatory effect of silybin on animal growth performance might be related to its regulatory mechanism on the intestine.

As one of the hallmarks of the acquired immune system, immunoglobulins have been rapidly and extensively explored and studied for their immunomodulatory and anti-inflammatory properties, and their levels correlate with the level of immunity in the body (33, 34). Studies have confirmed that Chinese herbal extracts can significantly improve the body’s immunity (35). In this study, silybin significantly increased serum IgA levels compared to controls, and high levels of addition can also significantly improve the IgG content. The results showed that the addition of silybin in the ration can improve the immunity level and enhance the disease resistance of Peking ducks. Inflammatory factors play an important role in the regulation of pathogenic infection and immune homeostasis. Interleukin-6 (IL-6) is an important inflammatory factor involved in a variety of inflammatory responses or inflammatory disease processes (36); Tumor necrosis factor-α (TNF-α) has a strong pro-inflammatory capacity and can establish an inflammatory response through activation of the MAPK and NF-κB cascade signaling pathways (37). We found that serum levels of IL-6 and TNF-α were significantly reduced by the addition of 1,600 mg/kg silybin to the ration, which indicates that silybin can improve the anti-inflammatory ability of the organism and achieve the effect of organismal defense. This is similar to previous studies, also confirming the anti-inflammatory properties of silybin (14, 38). Silybin has excellent antioxidant ability, maintaining the REDOX balance of the body by scavenging free radicals, activating the body’s antioxidant system and preventing the formation of lipid peroxides (13, 15, 27). We observed that a significant increase in serum levels of total superoxide dismutase (T-SOD) occurred in the group supplemented with 400 mg/kg of silybin in the ration, and the same finding was observed in another study (39). T-SOD is the most important and optimal free radical scavenger in organisms, protecting the organism from oxidative damage by specifically scavenging superoxide anion (40). Furthermore, by reducing the expression of inflammatory response markers, silybin can further regulate the expression activity of downstream genes/pathways, thereby inhibiting the expression of iNOS and regulating the redox state of the body (10, 41). Therefore, we hypothesized that the antioxidant capacity of silybin may be achieved by scavenging free radicals or anti-inflammation, while the incomplete representation of the results may be related to the sample ingestion time, ample source and other factors. The liver is the largest and most important metabolic organ, and the health status of the liver can be roughly understood through the content changes of liver enzymes such as ALT and AST in serum. The increase of liver enzymes in serum often indicates liver disease, bile duct disease and other diseases. Compared with the control group, the serum ALT content in the S1600 group in this study was significantly reduced, indicating that liver function was improved, which was consistent with the results of other studies. Compared with the control group, the serum levels of ALT and AST were significantly reduced in the NAFLD model group mice after silymarin treatment. The liver function of the mice was significantly improved (42). In addition, in a randomized controlled clinical trial in human medicine, it was found that the serum ALT and AST levels of patients with silymarin were reduced by 0.26 IU/mL and 0.53 IU/mL, respectively, both of which were statistically significant (43). Therefore, we believe that the addition of silybin to the ration can have a protective effect on the liver of Peking ducks.

To investigate the effects of silybin on physiological processes and metabolic pathways in Peking ducks, transcriptomic analysis was performed on the liver of 42-day-old Peking ducks. KEGG enrichment showed that the differentially expressed genes in the S1600 group and the control group were significantly up-regulated in the antigen processing and presentation pathways. In our results, a significant increase in the level of immunity occurred in the S1600 group, and the resistance to disease was strengthened. Processing and presentation of antigens is an important basis of acquired immunity. As an important immune organ of the body, liver plays an important role in antigen presentation. Disturbance of liver homeostasis can lead to a variety of liver diseases, such as autoimmune hepatitis and cirrhosis. Moreover, when the organism recognizes a pathogen or its own antigenic response, the cells in the liver can respond rapidly, exhibiting excellent antigen-presenting properties (44). We also observed that differentially expressed genes in the insulin resistance pathway were significantly downregulated in the S1600 and control groups. Insulin resistance is associated with many pathologic conditions of the liver, influenced by genetic and environmental factors, and is present in many metabolically related diseases (27, 45). What has been shown is that pro-inflammatory factors can induce the development of insulin resistance. A study found that the insulin resistance of mice was significantly improved after the application of neutralizing IL-6 antibody (46). Similarly, silybin treatment reduced the expression levels of TNF-α and NF-κB in high-fat mice, reducing body weight and insulin resistance (47). This is in agreement with our previous findings that the addition of 1,600 mg/kg silybin significantly reduced serum levels of the pro-inflammatory factors IL-6 and TNF-α.

## 5 Conclusion

In this experiment, we investigated the effects of feed supplementation with silybin on growth performance, serum inflammatory factors, immune levels, antioxidant levels, serum biochemistry and liver transcriptome of Peking ducks. It was discovered that the addition of 1,600 mg/kg of silybin could greatly enhance the growth performance of Peking ducks, improve their immune ability and reduce the inflammatory response. The transcriptome results suggest that it might be achieved by regulating antigen processing and presentation, amino acid metabolism and synthesis, and JAK–STAT pathways.

## Data availability statement

The data presented in the study are deposited in the National Center for Biotechnology Information repository, accession number PRJNA1027007. The original contributions presented in the study are publicly available. This data can be found here: https://www.ncbi.nlm.nih.gov/sra/PRJNA1027007.

## Ethics statement

The animal study was approved by the Animal Welfare and Animal Experimental Ethics Review Committee of China Agricultural University. The study was conducted in accordance with the local legislation and institutional requirements.

## Author contributions

ZZ: Formal analysis, Methodology, Project administration, Visualization, Writing – original draft. BS: Data curation, Formal analysis, Writing – review & editing. XL: Funding acquisition, Project administration, Resources, Writing – review & editing. YD: Investigation, Resources, Writing – review & editing. LL: Conceptualization, Writing – review & editing. ZX: Supervision, Writing – review & editing.

## Footnotes

^1^ http://deweylab.biostat.wisc.edu/rsem/
