# Supplementary material for: Effects of silybin supplementation on growth performance, serum indexes and liver transcriptome of Peking ducks
Source: Front Vet Sci. 2024 Jan 3;10:1325115. doi: 10.3389/fvets.2023.1325115 (PMC10795170; doi:10.3389/fvets.2023.1325115)
Supplement: Supplementary file 1 [file Table_1.DOCX]

Supplementary Material

**Supplementary Table 1.** Statistics of differentially expressed genes between S400 and C.

| Gene ID | Gene description | FC | Log2FC | Regulate |
| --- | --- | --- | --- | --- |
| ENSAPLG00000000942 | chromosome 22 open reading frame 39 [Source: HGNC Symbol; Acc: HGNC: 27012] | 0.357 | -1.484436499 | down |
| ENSAPLG00000001480 | - | 3.648 | 1.866909459 | up |
| ENSAPLG00000002641 | ELOVL fatty acid elongase 2 [Source: HGNC Symbol; Acc: HGNC: 14416] | 0.135 | -2.893732211 | down |
| ENSAPLG00000002703 | fatty acid synthase [Source: HGNC Symbol; Acc: HGNC: 3594] | 2.044 | 1.031079422 | up |
| ENSAPLG00000003848 | - | 0.403 | -1.312787988 | down |
| ENSAPLG00000004165 | - | 23.517 | 4.555625131 | up |
| ENSAPLG00000004498 | uridine phosphorylase 2 [Source: HGNC Symbol; Acc: HGNC:23061] | 3.074 | 1.620335568 | up |
| ENSAPLG00000004784 | ADAM metallopeptidase domain 22 [Source: HGNC Symbol; Acc: HGNC: 201] | 0.193 | -2.372660096 | down |
| ENSAPLG00000004865 | interleukin 15 [Source: HGNC Symbol; Acc: HGNC: 5977] | 0.355 | -1.492404871 | down |
| ENSAPLG00000005699 | t-SNARE domain containing 1 [Source: HGNC Symbol; Acc: HGNC: 26437] | 0.258 | -1.951857117 | down |
| ENSAPLG00000006602 | kelch like family member 14 [Source: HGNC Symbol; Acc: HGNC: 29266] | 0.193 | -2.372374028 | down |
| ENSAPLG00000007414 | - | 4.139 | 2.049117502 | up |
| ENSAPLG00000007512 | recombination signal binding protein for immunoglobulin kappa J region [Source: HGNC Symbol; Acc: HGNC:5724] | 2.082 | 1.058261117 | up |
| ENSAPLG00000008520 | solute carrier family 12 member 8 [Source: HGNC Symbol; Acc: HGNC:15595] | 7.719 | 2.948470721 | up |
| ENSAPLG00000008786 | hydrogen voltage gated channel 1 [Source: HGNC Symbol; Acc: HGNC:28240] | 3.317 | 1.729783896 | up |
| ENSAPLG00000009115 | transmembrane protein 171 [Source: HGNC Symbol; Acc: HGNC:27031] | 0.171 | -2.550337107 | down |
| ENSAPLG00000009934 | 5-methyltetrahydrofolate-homocysteine methyltransferase [Source: HGNC Symbol; Acc: HGNC:7468] | 0.464 | -1.107424601 | down |
| ENSAPLG00000010694 | phosphoserine phosphatase [Source: HGNC Symbol; Acc: HGNC:9577] | 2.039 | 1.027707943 | up |
| ENSAPLG00000011120 | solute carrier family 25 member 30 [Source: HGNC Symbol; Acc: HGNC:27371] | 9.497 | 3.247501818 | up |
| ENSAPLG00000011932 | - | 2.554 | 1.352898276 | up |
| ENSAPLG00000012373 | abhydrolase domain containing 14B [Source: HGNC Symbol; Acc: HGNC:28235] | 0.255 | -1.973703778 | down |
| ENSAPLG00000012590 | - | 2.357 | 1.237204985 | up |
| ENSAPLG00000013827 | abhydrolase domain containing 11 [Source: HGNC Symbol; Acc: HGNC:16407] | 2.737 | 1.452812791 | up |
| ENSAPLG00000013997 | MYC proto-oncogene, bHLH transcription factor [Source: HGNC Symbol; Acc: HGNC:7553] | 0.49 | -1.028098586 | down |
| ENSAPLG00000014350 | - | 0.425 | -1.233099842 | down |
| ENSAPLG00000016019 | - | 8.352 | 3.062101349 | up |
| ENSAPLG00000017053 | - | 0.441 | -1.180140321 | down |
| ENSAPLG00000017181 | - | 0.075 | -3.744654866 | down |
| ENSAPLG00000017233 | - | 0.37 | -1.432840799 | down |
| ENSAPLG00000017306 | - | 5.797 | 2.535230941 | up |
| ENSAPLG00000017309 | - | 273.457 | 8.095170387 | up |
| ENSAPLG00000018039 | - | 2.483 | 1.31218443 | up |
| ENSAPLG00000018197 | adhesion molecule with Ig like domain 1 [Source: HGNC Symbol; Acc: HGNC:20824] | 0.474 | -1.076394607 | down |
| ENSAPLG00000018735 | NOP10 ribonucleoprotein [Source: HGNC Symbol; Acc: HGNC:14378] | 0.447 | -1.160819354 | down |
| ENSAPLG00000021584 | - | 0.425 | -1.233646898 | down |
| ENSAPLG00000022346 | - | 0.191 | -2.38593015 | down |
| ENSAPLG00000022737 | - | 0.372 | -1.427706221 | down |
| ENSAPLG00000022790 | sulfite oxidase [Source: HGNC Symbol; Acc: HGNC:11460] | 0.382 | -1.388932957 | down |
| ENSAPLG00000022939 | lactate dehydrogenase D [Source: HGNC Symbol; Acc: HGNC:19708] | 0.379 | -1.400748306 | down |
| ENSAPLG00000022980 | - | 0.336 | -1.575353465 | down |
| ENSAPLG00000023946 | X-box binding protein 1 [Source: HGNC Symbol; Acc: HGNC:12801] | 2.349 | 1.231774236 | up |
| ENSAPLG00000024111 | - | 4.853 | 2.278992901 | up |
| ENSAPLG00000024173 | - | 0.197 | -2.342323712 | down |
| ENSAPLG00000024420 | DnaJ heat shock protein family (Hsp40) member C22 [Source: HGNC Symbol; Acc: HGNC:25802] | 0.346 | -1.530697837 | down |
| ENSAPLG00000024517 | - | 0.123 | -3.01765832 | down |
| ENSAPLG00000024664 | - | 0.337 | -1.56843857 | down |
| ENSAPLG00000026970 | - | 0.189 | -2.400621566 | down |
| ENSAPLG00000027336 | - | 2.411 | 1.269386341 | up |
| ENSAPLG00000027402 | - | 0.232 | -2.110217715 | down |
| ENSAPLG00000027433 | - | 3.241 | 1.696302924 | up |
| ENSAPLG00000027465 | serpin family G member 1 [Source: HGNC Symbol; Acc: HGNC:1228] | 0.477 | -1.067455852 | down |
| ENSAPLG00000028309 | - | 0.056 | -4.157403849 | down |
| ENSAPLG00000029065 | - | 0.278 | -1.849291524 | down |
| ENSAPLG00000029583 | - | 0.189 | -2.39990595 | down |
| MSTRG.11374 | - | 0.007 | -7.128546784 | down |
| MSTRG.11444 | - | 0.134 | -2.898112439 | down |
| MSTRG.11578 | - | 19.247 | 4.266581469 | up |
| MSTRG.12149 | - | 0.403 | -1.309509345 | down |
| MSTRG.12164 | - | 0.435 | -1.202568417 | down |
| MSTRG.12361 | - | 0.033 | -4.942970515 | down |
| MSTRG.12366 | - | 0.44 | -1.183624786 | down |
| MSTRG.12594 | - | 0.105 | -3.253183338 | down |
| MSTRG.12883 | - | 56.878 | 5.829791185 | up |
| MSTRG.1309 | - | 797.438 | 9.639229217 | up |
| MSTRG.13245 | - | 3.529 | 1.819257275 | up |
| MSTRG.13593 | - | 0.341 | -1.552596969 | down |
| MSTRG.13768 | - | 0.027 | -5.231326659 | down |
| MSTRG.13894 | - | 0.007 | -7.260649543 | down |
| MSTRG.14357 | - | 0.421 | -1.249182407 | down |
| MSTRG.1582 | - | 3.678 | 1.878853313 | up |
| MSTRG.6269 | - | 0.143 | -2.806059852 | down |

**Supplementary Table 2.** Statistics of differentially expressed genes between S1600 and C.

| Gene ID | Gene description | FC | Log2FC | Regulate |
| --- | --- | --- | --- | --- |
| ENSAPLG00000000588 | - | 2.686 | 1.425493859 | up |
| ENSAPLG00000000603 | - | 0.465 | -1.10486009 | down |
| ENSAPLG00000000658 | - | 2.046 | 1.032469404 | up |
| ENSAPLG00000001196 | fatty acid binding protein 1 [Source: HGNC Symbol; Acc: HGNC:3555] | 0.264 | -1.921356275 | down |
| ENSAPLG00000001467 | DnaJ heat shock protein family (Hsp40) member A4 [Source: HGNC Symbol; Acc: HGNC:14885] | 2.674 | 1.419264511 | up |
| ENSAPLG00000001908 | protein phosphatase 1 regulatory subunit 3C [Source: HGNC Symbol; Acc: HGNC:9293] | 0.264 | -1.91895772 | down |
| ENSAPLG00000001969 | cysteine and serine rich nuclear protein 1 [Source: HGNC Symbol; Acc: HGNC:14300] | 2.716 | 1.441583425 | up |
| ENSAPLG00000001977 | complement C3a receptor 1 [Source: HGNC Symbol; Acc: HGNC:1319] | 2.625 | 1.392059252 | up |
| ENSAPLG00000002110 | DnaJ heat shock protein family (Hsp40) member B5 [Source: HGNC Symbol; Acc: HGNC:14887] | 2.497 | 1.3203084 | up |
| ENSAPLG00000002303 | stanniocalcin 2 [Source: HGNC Symbol; Acc: HGNC:11374] | 3.612 | 1.852694638 | up |
| ENSAPLG00000002641 | ELOVL fatty acid elongase 2 [Source: HGNC Symbol; Acc: HGNC:14416] | 0.417 | -1.261306223 | down |
| ENSAPLG00000002703 | fatty acid synthase [Source: HGNC Symbol; Acc: HGNC:3594] | 4.127 | 2.045004692 | up |
| ENSAPLG00000002776 | death associated protein kinase 2 [Source: HGNC Symbol; Acc: HGNC:2675] | 2.31 | 1.20792889 | up |
| ENSAPLG00000002915 | actin alpha 2, smooth muscle [Source: HGNC Symbol; Acc: HGNC:130] | 2.666 | 1.41453393 | up |
| ENSAPLG00000002963 | zinc finger and BTB domain containing 16 [Source: HGNC Symbol; Acc: HGNC:12930] | 6.999 | 2.80715817 | up |
| ENSAPLG00000002995 | complement C1q B chain [Source: HGNC Symbol; Acc: HGNC:1242] | 2.043 | 1.030581655 | up |
| ENSAPLG00000003320 | serum response factor [Source: HGNC Symbol; Acc: HGNC:11291] | 2.768 | 1.469060199 | up |
| ENSAPLG00000003607 | DNA methyltransferase 3 beta [Source: HGNC Symbol; Acc: HGNC:2979] | 0.119 | -3.074016851 | down |
| ENSAPLG00000003627 | FKBP prolyl isomerase 5 [Source: HGNC Symbol; Acc: HGNC:3721] | 3.06 | 1.613614194 | up |
| ENSAPLG00000003801 | zyxin [Source: HGNC Symbol; Acc:HGNC:13200] | 2.416 | 1.272750145 | up |
| ENSAPLG00000003835 | acyl-CoA thioesterase 11 [Source: HGNC Symbol; Acc: HGNC:18156] | 2.951 | 1.561305852 | up |
| ENSAPLG00000003908 | thrombospondin 1 [Source: HGNC Symbol; Acc: HGNC:11785] | 2.995 | 1.582405926 | up |
| ENSAPLG00000004284 | 2-phosphoxylose phosphatase 1 [Source: HGNC Symbol; Acc: HGNC:26303] | 0.427 | -1.226383459 | down |
| ENSAPLG00000004369 | regulatory factor X2 [Source: HGNC Symbol; Acc: HGNC:9983] | 2.659 | 1.411086476 | up |
| ENSAPLG00000004482 | - | 2.156 | 1.10806767 | up |
| ENSAPLG00000004498 | uridine phosphorylase 2 [Source: HGNC Symbol; Acc: HGNC:23061] | 11.548 | 3.529508782 | up |
| ENSAPLG00000004546 | - | 2.813 | 1.491909651 | up |
| ENSAPLG00000004576 | ubiquitin specific peptidase 44 [Source: HGNC Symbol; Acc: HGNC:20064] | 2.214 | 1.146890105 | up |
| ENSAPLG00000004592 | DnaJ heat shock protein family (Hsp40) member C12 [Source: HGNC Symbol; Acc: HGNC:28908] | 5.294 | 2.404417049 | up |
| ENSAPLG00000004813 | - | 2.398 | 1.262114496 | up |
| ENSAPLG00000005104 | heat shock protein family H (Hsp110) member 1 [Source: HGNC Symbol; Acc: HGNC:16969] | 2.324 | 1.216668439 | up |
| ENSAPLG00000005132 | microtubule associated protein 9 [Source: HGNC Symbol; Acc: HGNC:26118] | 0.469 | -1.09155497 | down |
| ENSAPLG00000005188 | RasGEF domain family member 1A [Source: HGNC Symbol; Acc: HGNC:24246] | 2.383 | 1.252770423 | up |
| ENSAPLG00000005372 | - | 2.649 | 1.405517974 | up |
| ENSAPLG00000005388 | phosphoserine aminotransferase 1 [Source: HGNC Symbol; Acc: HGNC:19129] | 3.104 | 1.634283786 | up |
| ENSAPLG00000005506 | - | 2.692 | 1.428864841 | up |
| ENSAPLG00000005578 | - | 0.263 | -1.927250275 | down |
| ENSAPLG00000005672 | four and a half LIM domains 5 [Source: HGNC Symbol; Acc: HGNC:17371] | 2.51 | 1.327862469 | up |
| ENSAPLG00000005745 | perilipin 2 [Source: HGNC Symbol; Acc: HGNC:248] | 2.679 | 1.421756601 | up |
| ENSAPLG00000006322 | - | 0.239 | -2.064864215 | down |
| ENSAPLG00000006602 | kelch like family member 14 [Source: HGNC Symbol; Acc: HGNC:29266] | 0.485 | -1.044527093 | down |
| ENSAPLG00000006947 | thyrotropin releasing hormone receptor [Source: HGNC Symbol; Acc: HGNC:12299] | 0.438 | -1.189984485 | down |
| ENSAPLG00000007165 | papilin, proteoglycan like sulfated glycoprotein [Source: HGNC Symbol; Acc: HGNC:19262] | 0.476 | -1.07016117 | down |
| ENSAPLG00000007167 | cyclin Y like 1 [Source: HGNC Symbol; Acc: HGNC:26868] | 3.633 | 1.861068784 | up |
| ENSAPLG00000007240 | phosphodiesterase 4B [Source: HGNC Symbol; Acc: HGNC:8781] | 3.367 | 1.751528593 | up |
| ENSAPLG00000007495 | Kruppel like factor 3 [Source: HGNC Symbol; Acc: HGNC:16516] | 2.187 | 1.128886822 | up |
| ENSAPLG00000007512 | recombination signal binding protein for immunoglobulin kappa J region [Source: HGNC Symbol; Acc: HGNC:5724] | 3.021 | 1.595053706 | up |
| ENSAPLG00000007797 | - | 3.629 | 1.859456975 | up |
| ENSAPLG00000007890 | abhydrolase domain containing 5 [Source: HGNC Symbol; Acc: HGNC:21396] | 2.476 | 1.307985355 | up |
| ENSAPLG00000007914 | - | 2.897 | 1.534678807 | up |
| ENSAPLG00000008156 | ethanolamine-phosphate phospho-lyase [Source: HGNC Symbol; Acc: HGNC:14404] | 3.23 | 1.691755359 | up |
| ENSAPLG00000008235 | - | 39.156 | 5.291154749 | up |
| ENSAPLG00000008578 | - | 2.145 | 1.101034766 | up |
| ENSAPLG00000008655 | - | 2.688 | 1.426484055 | up |
| ENSAPLG00000008775 | THUMP domain containing 2 [Source: HGNC Symbol; Acc: HGNC:14890] | 2.281 | 1.189679395 | up |
| ENSAPLG00000008961 | cytochrome b-245 beta chain [Source: HGNC Symbol; Acc: HGNC:2578] | 2.712 | 1.439276701 | up |
| ENSAPLG00000009032 | complement C7 [Source: HGNC Symbol; Acc: HGNC:1346] | 2.411 | 1.269863922 | up |
| ENSAPLG00000009106 | phosphoribosyl pyrophosphate amidotransferase [Source: HGNC Symbol; Acc: HGNC:9238] | 2.131 | 1.091776085 | up |
| ENSAPLG00000009413 | solute carrier family 16 member 6 [Source: HGNC Symbol; Acc: HGNC:10927] | 3.753 | 1.907959524 | up |
| ENSAPLG00000009439 | phosphoribosyl aminoimidazole carboxylase and phosphoribosyl aminoimidazole succinocarboxamide synthase [Source: HGNC Symbol; Acc: HGNC:8587] | 2.757 | 1.463068786 | up |
| ENSAPLG00000009474 | - | 2.03 | 1.021601013 | up |
| ENSAPLG00000009589 | cadherin related family member 2 [Source: HGNC Symbol; Acc: HGNC:18231] | 2.581 | 1.368033444 | up |
| ENSAPLG00000009685 | - | 2.179 | 1.123589301 | up |
| ENSAPLG00000009771 | methylenetetrahydrofolate reductase [Source: HGNC Symbol; Acc: HGNC:7436] | 0.382 | -1.388253636 | down |
| ENSAPLG00000009896 | dual specificity phosphatase 8 [Source: HGNC Symbol; Acc: HGNC:3074] | 4.67 | 2.223502056 | up |
| ENSAPLG00000009922 | PDZ and LIM domain 3 [Source: HGNC Symbol; Acc: HGNC:20767] | 5.238 | 2.3889951 | up |
| ENSAPLG00000009928 | vinculin [Source: HGNC Symbol; Acc: HGNC:12665] | 2.649 | 1.405209099 | up |
| ENSAPLG00000009975 | hedgehog interacting protein [Source: HGNC Symbol; Acc: HGNC:14866] | 2.469 | 1.303773776 | up |
| ENSAPLG00000010039 | - | 3.92 | 1.970948766 | up |
| ENSAPLG00000010161 | arrestin domain containing 4 [Source: HGNC Symbol; Acc: HGNC:28087] | 2.047 | 1.03381826 | up |
| ENSAPLG00000010356 | tetratricopeptide repeat and ankyrin repeat containing 1 [Source: HGNC Symbol; Acc: HGNC:29011] | 2.227 | 1.155294392 | up |
| ENSAPLG00000010455 | - | 3.042 | 1.605141454 | up |
| ENSAPLG00000010667 | heat shock protein family A (Hsp70) member 8 [Source: HGNC Symbol; Acc: HGNC:5241] | 2.236 | 1.160925826 | up |
| ENSAPLG00000010761 | filamin A interacting protein 1 like [Source: HGNC Symbol; Acc: HGNC:24589] | 3.359 | 1.748157714 | up |
| ENSAPLG00000010946 | xanthine dehydrogenase [Source: HGNC Symbol; Acc: HGNC:12805] | 2.02 | 1.014557868 | up |
| ENSAPLG00000011020 | ERBB receptor feedback inhibitor 1 [Source: HGNC Symbol; Acc: HGNC:18185] | 3.552 | 1.82852807 | up |
| ENSAPLG00000011053 | GLI pathogenesis related 2 [Source: HGNC Symbol; Acc: HGNC:18007] | 3.745 | 1.904915682 | up |
| ENSAPLG00000011120 | solute carrier family 25 member 30 [Source: HGNC Symbol; Acc: HGNC:27371] | 7.194 | 2.846712619 | up |
| ENSAPLG00000011285 | ATPase Na+/K+ transporting subunit alpha 1 [Source: HGNC Symbol; Acc: HGNC:799] | 2.768 | 1.468781219 | up |
| ENSAPLG00000011426 | chromosome 21 open reading frame 62 [Source: HGNC Symbol; Acc: HGNC:1305] | 5.147 | 2.363704485 | up |
| ENSAPLG00000011656 | FLVCR heme transporter 2 [Source: HGNC Symbol; Acc: HGNC:20105] | 2.239 | 1.163032362 | up |
| ENSAPLG00000011744 | RAB20, member RAS oncogene family [Source: HGNC Symbol; Acc: HGNC:18260] | 2.018 | 1.012627419 | up |
| ENSAPLG00000011932 | - | 2.144 | 1.100450708 | up |
| ENSAPLG00000012199 | transforming growth factor beta 3 [Source: HGNC Symbol; Acc: HGNC:11769] | 3.022 | 1.595647351 | up |
| ENSAPLG00000012373 | abhydrolase domain containing 14B [Source: HGNC Symbol; Acc: HGNC:28235] | 0.35 | -1.513602284 | down |
| ENSAPLG00000012382 | - | 2.508 | 1.326769562 | up |
| ENSAPLG00000012402 | TLE family member 1, transcriptional corepressor [Source: HGNC Symbol; Acc: HGNC:11837] | 2.729 | 1.448441975 | up |
| ENSAPLG00000012488 | ITPR interacting domain containing 2 [Source: HGNC Symbol; Acc: HGNC:11319] | 3.566 | 1.834164552 | up |
| ENSAPLG00000012495 | - | 2.347 | 1.231075647 | up |
| ENSAPLG00000012578 | solute carrier family 41 member 3 [Source: HGNC Symbol; Acc: HGNC:31046] | 2.426 | 1.278856244 | up |
| ENSAPLG00000012624 | cathepsin S [Source: HGNC Symbol; Acc: HGNC:2545] | 2.459 | 1.298361944 | up |
| ENSAPLG00000012627 | CECR2 histone acetyl-lysine reader [Source: HGNC Symbol; Acc: HGNC:1840] | 0.361 | -1.471281071 | down |
| ENSAPLG00000012991 | p53-induced death domain protein 1 [Source: HGNC Symbol; Acc: HGNC:16491] | 0.477 | -1.066579524 | down |
| ENSAPLG00000013355 | cache domain containing 1 [Source: HGNC Symbol; Acc: HGNC:29314] | 2.645 | 1.403409645 | up |
| ENSAPLG00000013386 | lipin 1 [Source: HGNC Symbol; Acc: HGNC:13345] | 3.459 | 1.790387407 | up |
| ENSAPLG00000013513 | four and a half LIM domains 2 [Source: HGNC Symbol; Acc: HGNC:3703] | 2.005 | 1.003836602 | up |
| ENSAPLG00000013843 | cAMP responsive element modulator [Source: HGNC Symbol; Acc: HGNC:2352] | 3.821 | 1.933841434 | up |
| ENSAPLG00000013859 | aminocarboxymuconate semialdehyde decarboxylase [Source: HGNC Symbol; Acc: HGNC:19288] | 2.889 | 1.530389986 | up |
| ENSAPLG00000014313 | prostaglandin-endoperoxide synthase 1 [Source: HGNC Symbol; Acc: HGNC:9604] | 2.295 | 1.19850617 | up |
| ENSAPLG00000014350 | - | 0.488 | -1.034191277 | down |
| ENSAPLG00000014414 | - | 0.419 | -1.255193449 | down |
| ENSAPLG00000014490 | GTP binding protein overexpressed in skeletal muscle [Source: HGNC Symbol; Acc: HGNC:4234] | 2.404 | 1.26551474 | up |
| ENSAPLG00000014661 | acyl-CoA synthetase long chain family member 5 [Source: HGNC Symbol; Acc: HGNC:16526] | 2.524 | 1.335743264 | up |
| ENSAPLG00000014681 | serine and arginine rich splicing factor 5 [Source: HGNC Symbol; Acc: HGNC:10787] | 0.486 | -1.040706375 | down |
| ENSAPLG00000014697 | NADPH oxidase activator 1 [Source: HGNC Symbol; Acc: HGNC:10668] | 2.854 | 1.512804758 | up |
| ENSAPLG00000014782 | pleckstrin 2 [Source: HGNC Symbol; Acc: HGNC:19238] | 11.595 | 3.535393491 | up |
| ENSAPLG00000015189 | abhydrolase domain containing 17B [Source: HGNC Symbol; Acc: HGNC:24278] | 3.299 | 1.72183157 | up |
| ENSAPLG00000015226 | proline-serine-threonine phosphatase interacting protein 1 [Source: HGNC Symbol; Acc: HGNC:9580] | 3.029 | 1.598889586 | up |
| ENSAPLG00000015602 | arginase 2 [Source: HGNC Symbol; Acc: HGNC:664] | 13.476 | 3.752279372 | up |
| ENSAPLG00000015618 | beta-1,4-galactosyltransferase 5 [Source: HGNC Symbol; Acc: HGNC:928] | 3.011 | 1.59019926 | up |
| ENSAPLG00000015665 | stearoyl-CoA desaturase [Source: HGNC Symbol; Acc: HGNC:10571] | 2.844 | 1.50770713 | up |
| ENSAPLG00000015678 | pyruvate dehydrogenase kinase 4 [Source: HGNC Symbol; Acc: HGNC:8812] | 3.301 | 1.722747058 | up |
| ENSAPLG00000015733 | FKBP prolyl isomerase 10 [Source: HGNC Symbol; Acc: HGNC:18169] | 0.123 | -3.024431066 | down |
| ENSAPLG00000015814 | neuroligin 3 [Source: HGNC Symbol; Acc: HGNC:14289] | 3.258 | 1.703785571 | up |
| ENSAPLG00000015834 | RUN and FYVE domain containing 2 [Source: HGNC Symbol; Acc: HGNC:19761] | 0.475 | -1.07405533 | down |
| ENSAPLG00000016057 | tubulin tyrosine ligase like 7 [Source: HGNC Symbol; Acc: HGNC:26242] | 2.459 | 1.298213895 | up |
| ENSAPLG00000016067 | - | 2.123 | 1.086015463 | up |
| ENSAPLG00000016293 | - | 0.041 | -4.617279496 | down |
| ENSAPLG00000016324 | FTO alpha-ketoglutarate dependent dioxygenase [Source: HGNC Symbol; Acc: HGNC:24678] | 0.426 | -1.23248075 | down |
| ENSAPLG00000016454 | Pim-3 proto-oncogene, serine/threonine kinase [Source: HGNC Symbol; Acc: HGNC:19310] | 0.382 | -1.386675012 | down |
| ENSAPLG00000017181 | - | 0.13 | -2.941949291 | down |
| ENSAPLG00000017196 | solute carrier family 38 member 2 [Source: HGNC Symbol; Acc: HGNC:13448] | 3.273 | 1.710440927 | up |
| ENSAPLG00000017218 | chromosome 6 open reading frame 132 [Source: HGNC Symbol; Acc: HGNC:21288] | 0.268 | -1.900965016 | down |
| ENSAPLG00000017233 | - | 2.077 | 1.054587151 | up |
| ENSAPLG00000017306 | - | 5.892 | 2.55883884 | up |
| ENSAPLG00000017309 | - | 391.454 | 8.612697352 | up |
| ENSAPLG00000017485 | transmembrane protein 53 [Source: HGNC Symbol; Acc: HGNC:26186] | 0.441 | -1.180346813 | down |
| ENSAPLG00000017600 | - | 2.854 | 1.513061771 | up |
| ENSAPLG00000017806 | ankyrin repeat domain 9 [Source: HGNC Symbol; Acc: HGNC:20096] | 2.262 | 1.177452195 | up |
| ENSAPLG00000017871 | - | 0.332 | -1.589233377 | down |
| ENSAPLG00000018151 | - | 0.047 | -4.415499106 | down |
| ENSAPLG00000018200 | oxidative stress induced growth inhibitor 1 [Source: HGNC Symbol; Acc: HGNC:30093] | 0.243 | -2.039928991 | down |
| ENSAPLG00000018235 | coenzyme Q10A [Source: HGNC Symbol; Acc: HGNC:26515] | 2.02 | 1.014580706 | up |
| ENSAPLG00000018995 | - | 2.548 | 1.349144207 | up |
| ENSAPLG00000019280 | - | 0.491 | -1.025528454 | down |
| ENSAPLG00000019495 | - | 0.139 | -2.842431787 | down |
| ENSAPLG00000019896 | - | 3.748 | 1.906219449 | up |
| ENSAPLG00000020045 | one cut homeobox 1 [Source: HGNC Symbol; Acc: HGNC:8138] | 0.467 | -1.097951246 | down |
| ENSAPLG00000020095 | - | 0.256 | -1.96778276 | down |
| ENSAPLG00000020285 | - | 2.088 | 1.061832305 | up |
| ENSAPLG00000020408 | Gse1 coiled-coil protein [Source: HGNC Symbol; Acc: HGNC:28979] | 0.415 | -1.269755425 | down |
| ENSAPLG00000020610 | - | 2.219 | 1.149872575 | up |
| ENSAPLG00000020771 | - | 0.444 | -1.172193607 | down |
| ENSAPLG00000021074 | - | 0.488 | -1.034567261 | down |
| ENSAPLG00000021187 | retinoic acid receptor alpha [Source: HGNC Symbol; Acc: HGNC:9864] | 2.025 | 1.017978222 | up |
| ENSAPLG00000021584 | - | 0.464 | -1.107396919 | down |
| ENSAPLG00000021865 | MOB kinase activator 3A [Source: HGNC Symbol; Acc: HGNC:29802] | 2.157 | 1.108710723 | up |
| ENSAPLG00000021872 | - | 0.315 | -1.667195922 | down |
| ENSAPLG00000022020 | - | 4.339 | 2.117434322 | up |
| ENSAPLG00000022169 | - | 0.202 | -2.305634874 | down |
| ENSAPLG00000022187 | - | 0.434 | -1.203006432 | down |
| ENSAPLG00000022252 | - | 0.424 | -1.237191929 | down |
| ENSAPLG00000022276 | MAPK interacting serine/threonine kinase 2 [Source: HGNC Symbol; Acc: HGNC:7111] | 2.409 | 1.268266833 | up |
| ENSAPLG00000022295 | TNF superfamily member 10 [Source: HGNC Symbol; Acc: HGNC:11925] | 0.384 | -1.379868033 | down |
| ENSAPLG00000022737 | - | 0.457 | -1.130654623 | down |
| ENSAPLG00000023048 | - | 5.394 | 2.431245494 | up |
| ENSAPLG00000023238 | - | 2.22 | 1.150717835 | up |
| ENSAPLG00000023455 | RAB27B, member RAS oncogene family [Source: HGNC Symbol; Acc: HGNC:9767] | 2.257 | 1.174254133 | up |
| ENSAPLG00000023484 | TIMP metallopeptidase inhibitor 3 [Source: HGNC Symbol; Acc: HGNC:11822] | 2.478 | 1.309186119 | up |
| ENSAPLG00000023956 | - | 3.208 | 1.681623514 | up |
| ENSAPLG00000024016 | - | 0.375 | -1.415741827 | down |
| ENSAPLG00000024111 | - | 4.126 | 2.044639215 | up |
| ENSAPLG00000024305 | GLI family zinc finger 1 [Source: HGNC Symbol; Acc: HGNC:4317] | 0.26 | -1.943838762 | down |
| ENSAPLG00000024420 | DnaJ heat shock protein family (Hsp40) member C22 [Source: HGNC Symbol; Acc: HGNC:25802] | 0.392 | -1.351078747 | down |
| ENSAPLG00000024542 | zinc finger CCCH-type containing 12A [Source: HGNC Symbol; Acc: HGNC:26259] | 2.235 | 1.160148513 | up |
| ENSAPLG00000024664 | - | 0.235 | -2.08773514 | down |
| ENSAPLG00000024792 | protein phosphatase 1 regulatory subunit 3G [Source: HGNC Symbol; Acc: HGNC:14945] | 4.144 | 2.051124982 | up |
| ENSAPLG00000024808 | - | 2.198 | 1.136359288 | up |
| ENSAPLG00000024935 | growth arrest and DNA damage inducible gamma [Source: HGNC Symbol; Acc: HGNC:4097] | 2.068 | 1.048222277 | up |
| ENSAPLG00000025519 | myosin light chain 9 [Source: HGNC Symbol; Acc: HGNC:15754] | 2.35 | 1.232397614 | up |
| ENSAPLG00000025865 | acylphosphatase 1 [Source: HGNC Symbol; Acc: HGNC:179] | 0.356 | -1.489208467 | down |
| ENSAPLG00000026069 | - | 0.445 | -1.168568682 | down |
| ENSAPLG00000026143 | - | 2.574 | 1.364142422 | up |
| ENSAPLG00000026260 | - | 5.511 | 2.462408906 | up |
| ENSAPLG00000026346 | mediator complex subunit 11 [Source: HGNC Symbol; Acc: HGNC:32687] | 0.417 | -1.262765952 | down |
| ENSAPLG00000026479 | deoxyribonuclease 2 beta [Source: HGNC Symbol; Acc: HGNC:28875] | 2.256 | 1.173856626 | up |
| ENSAPLG00000026690 | TraB domain containing 2A [Source: HGNC Symbol; Acc: HGNC:27013] | 3.906 | 1.965867964 | up |
| ENSAPLG00000026694 | - | 2.197 | 1.135380774 | up |
| ENSAPLG00000026879 | nocturnin [Source: HGNC Symbol; Acc: HGNC:14254] | 4.831 | 2.272238029 | up |
| ENSAPLG00000027216 | - | 0.175 | -2.513790121 | down |
| ENSAPLG00000027336 | - | 2.762 | 1.465908411 | up |
| ENSAPLG00000027345 | tubulin beta 2B class IIb [Source: HGNC Symbol; Acc: HGNC:30829] | 2.047 | 1.033358521 | up |
| ENSAPLG00000027479 | - | 2.723 | 1.444959774 | up |
| ENSAPLG00000027519 | - | 2.374 | 1.247316612 | up |
| ENSAPLG00000027590 | - | 0.417 | -1.261914538 | down |
| ENSAPLG00000027827 | desmin [Source: HGNC Symbol; Acc: HGNC:2770] | 2.021 | 1.015287337 | up |
| ENSAPLG00000028561 | - | 2.635 | 1.397983112 | up |
| ENSAPLG00000028616 | - | 0.402 | -1.31395259 | down |
| ENSAPLG00000028889 | - | 4.632 | 2.211676904 | up |
| ENSAPLG00000029065 | - | 0.408 | -1.293120284 | down |
| ENSAPLG00000029075 | - | 2.216 | 1.147861095 | up |
| ENSAPLG00000029165 | BLOC-1 related complex subunit 6 [Source: HGNC Symbol; Acc: HGNC:25939] | 0.384 | -1.382643009 | down |
| ENSAPLG00000029441 | - | 3.257 | 1.703657984 | up |
| ENSAPLG00000029583 | - | 0.254 | -1.974446833 | down |
| ENSAPLG00000029672 | - | 3.236 | 1.694364677 | up |
| ENSAPLG00000029790 | - | 2.127 | 1.089085037 | up |
| ENSAPLG00000029863 | complement C1q C chain [Source: HGNC Symbol; Acc: HGNC:1245] | 2.232 | 1.158050226 | up |
| ENSAPLG00000030106 | - | 2.164 | 1.113624477 | up |
| ENSAPLG00000030662 | - | 2.04 | 1.028224364 | up |
| ENSAPLG00000030665 | - | 0.404 | -1.306365819 | down |
| ENSAPLG00000030874 | - | 2.735 | 1.451519566 | up |
| ENSAPLG00000031127 | - | 0.131 | -2.936676545 | down |
| ENSAPLG00000031136 | - | 0.42 | -1.251575403 | down |
| ENSAPLG00000031161 | - | 2.659 | 1.410650985 | up |
| ENSAPLG00000031181 | - | 0.491 | -1.02550165 | down |
| MSTRG.10398 | - | 3.014 | 1.591512916 | up |
| MSTRG.10545 | - | 2.287 | 1.193279257 | up |
| MSTRG.10972 | - | 0.435 | -1.201188322 | down |
| MSTRG.11056 | - | 0.136 | -2.87977405 | down |
| MSTRG.11059 | - | 5.377 | 2.426776906 | up |
| MSTRG.11136 | - | 0.412 | -1.277896656 | down |
| MSTRG.11410 | - | 0.106 | -3.235170023 | down |
| MSTRG.11444 | - | 0.161 | -2.63885368 | down |
| MSTRG.11617 | - | 0.272 | -1.879600696 | down |
| MSTRG.11639 | - | 2.386 | 1.254857677 | up |
| MSTRG.11659 | - | 0.18 | -2.475080232 | down |
| MSTRG.12205 | - | 0.2 | -2.324927667 | down |
| MSTRG.12292 | - | 5.854 | 2.549514971 | up |
| MSTRG.12406 | - | 3.428 | 1.777325454 | up |
| MSTRG.126 | - | 10.51 | 3.393741258 | up |
| MSTRG.12975 | - | 8.714 | 3.123415264 | up |
| MSTRG.13004 | - | 0.365 | -1.453701015 | down |
| MSTRG.13086 | - | 0.341 | -1.551317681 | down |
| MSTRG.13443 | - | 2.932 | 1.551773338 | up |
| MSTRG.13444 | - | 2.297 | 1.199854755 | up |
| MSTRG.13460 | - | 0.407 | -1.297920533 | down |
| MSTRG.13497 | - | 0.36 | -1.472184975 | down |
| MSTRG.13593 | - | 0.359 | -1.47851572 | down |
| MSTRG.13599 | - | 0.001 | -9.929952932 | down |
| MSTRG.13671 | - | 0.257 | -1.958256038 | down |
| MSTRG.13761 | - | 0.434 | -1.203789887 | down |
| MSTRG.13891 | - | 0.276 | -1.856977557 | down |
| MSTRG.13954 | - | 0.15 | -2.733938372 | down |
| MSTRG.14108 | - | 0.355 | -1.493051051 | down |
| MSTRG.14229 | - | 4.47 | 2.160389141 | up |
| MSTRG.1661 | - | 2.358 | 1.237589332 | up |
| MSTRG.2603 | - | 2.369 | 1.244508281 | up |
| MSTRG.3343 | - | 0.381 | -1.390348499 | down |
| MSTRG.343 | - | 2.034 | 1.024135034 | up |
| MSTRG.3825 | - | 0.23 | -2.122600258 | down |
| MSTRG.3956 | - | 0.427 | -1.229010395 | down |
| MSTRG.3967 | - | 3.084 | 1.624858589 | up |
| MSTRG.3968 | - | 2.841 | 1.506644163 | up |
| MSTRG.4823 | - | 0.252 | -1.990009885 | down |
| MSTRG.5202 | - | 2.563 | 1.3578448 | up |
| MSTRG.5488 | - | 2.092 | 1.065174526 | up |
| MSTRG.5959 | - | 6.457 | 2.690948392 | up |
| MSTRG.6128 | - | 3.178 | 1.667904023 | up |
| MSTRG.620 | - | 0.272 | -1.876759712 | down |
| MSTRG.621 | - | 0.443 | -1.175412738 | down |
| MSTRG.6598 | - | 0.459 | -1.124761832 | down |
| MSTRG.6733 | - | 0.286 | -1.804626711 | down |
| MSTRG.6789 | - | 0.069 | -3.855377675 | down |
| MSTRG.7165 | - | 2.111 | 1.077946563 | up |
| MSTRG.7423 | - | 4.513 | 2.173990755 | up |
| MSTRG.7549 | - | 0.312 | -1.680769025 | down |
| MSTRG.9701 | - | 0.299 | -1.742924607 | down |
| MSTRG.9982 | - | 0.257 | -1.96149757 | down |

**Supplementary Table 3.** KEGG enrichment analysis of DEGs in S400 and C groups.

| Pathway ID | Description | P value | P adjust | Gene IDs |
| --- | --- | --- | --- | --- |
| map00983 | Drug metabolism - other enzymes | 0.002259327 | 0.26434122 | ENSAPLG00000029583\|MSTRG.13245\|ENSAPLG00000004498 |
| map01040 | Biosynthesis of unsaturated fatty acids | 0.006588553 | 0.385430367 | MSTRG.13593\|ENSAPLG00000002641 |
| map00270 | Cysteine and methionine metabolism | 0.013626759 | 0.398582702 | ENSAPLG00000009934\|ENSAPLG00000016019 |
| map05204 | Chemical carcinogenesis | 0.160477049 | 0.481431147 | ENSAPLG00000018039 |
| map05163 | Human cytomegalovirus infection | 0.19877202 | 0.484506798 | ENSAPLG00000013997\|MSTRG.12361 |
| map00400 | Phenylalanine, tyrosine and tryptophan biosynthesis | 0.020740814 | 0.485335045 | ENSAPLG00000016019 |
| map04918 | Thyroid hormone synthesis | 0.220082989 | 0.48584358 | ENSAPLG00000001480 |
| map00380 | Tryptophan metabolism | 0.012624388 | 0.492351132 | ENSAPLG00000018039\|ENSAPLG00000016019 |
| map04061 | Viral protein interaction with cytokine and cytokine receptor | 0.206279012 | 0.492543764 | ENSAPLG00000027336 |
| map04330 | Notch signaling pathway | 0.19788195 | 0.492599748 | ENSAPLG00000007512 |
| map05221 | Acute myeloid leukemia | 0.236337323 | 0.493776192 | ENSAPLG00000013997 |
| map04391 | Hippo signaling pathway - fly | 0.241681268 | 0.496082603 | ENSAPLG00000013997 |
| map05133 | Pertussis | 0.233651522 | 0.49704051 | ENSAPLG00000027465 |
| map04978 | Mineral absorption | 0.157529935 | 0.4981352 | ENSAPLG00000001480 |
| map04973 | Carbohydrate digestion and absorption | 0.157529935 | 0.4981352 | ENSAPLG00000001480 |
| map04060 | Cytokine-cytokine receptor interaction | 0.183619829 | 0.499616743 | ENSAPLG00000004865\|ENSAPLG00000027336 |
| map05203 | Viral carcinogenesis | 0.153937599 | 0.500297197 | ENSAPLG00000007512\|MSTRG.12361 |
| map05332 | Graft-versus-host disease | 0.230956461 | 0.500405665 | MSTRG.12361 |
| map04913 | Ovarian steroidogenesis | 0.189397813 | 0.503626002 | ENSAPLG00000018039 |
| map00980 | Metabolism of xenobiotics by cytochrome P450 | 0.121363615 | 0.507126535 | ENSAPLG00000018039 |
| map00970 | Aminoacyl-tRNA biosynthesis | 0.195063623 | 0.507165419 | ENSAPLG00000024173 |
| map00280 | Valine, leucine and isoleucine degradation | 0.195063623 | 0.507165419 | ENSAPLG00000016019 |
| map05213 | Endometrial cancer | 0.217341157 | 0.508578306 | ENSAPLG00000013997 |
| map04979 | Cholesterol metabolism | 0.217341157 | 0.508578306 | ENSAPLG00000004165 |
| map05230 | Central carbon metabolism in cancer | 0.217341157 | 0.508578306 | ENSAPLG00000013997 |
| map05167 | Kaposi sarcoma-associated herpesvirus infection | 0.152722755 | 0.510530353 | ENSAPLG00000013997\|MSTRG.12361 |
| map04940 | Type I diabetes mellitus | 0.254881251 | 0.514157007 | MSTRG.12361 |
| map04672 | Intestinal immune network for IgA production | 0.083525426 | 0.514340783 | ENSAPLG00000004865\|ENSAPLG00000027336 |
| map00250 | Alanine, aspartate and glutamate metabolism | 0.180825715 | 0.516014844 | ENSAPLG00000016019 |
| map05219 | Bladder cancer | 0.180825715 | 0.516014844 | ENSAPLG00000013997 |
| map00230 | Purine metabolism | 0.119619451 | 0.518350955 | ENSAPLG00000029583\|MSTRG.13245 |
| map04961 | Endocrine and other factor-regulated calcium reabsorption | 0.177948648 | 0.520499794 | ENSAPLG00000001480 |
| map00260 | Glycine, serine and threonine metabolism | 0.151605247 | 0.521700407 | ENSAPLG00000010694 |
| map04260 | Cardiac muscle contraction | 0.267855892 | 0.522318989 | ENSAPLG00000001480 |
| map05220 | Chronic myeloid leukemia | 0.265278809 | 0.526061367 | ENSAPLG00000013997 |
| map04350 | TGF-beta signaling pathway | 0.293144168 | 0.527659502 | ENSAPLG00000013997 |
| map04919 | Thyroid hormone signaling pathway | 0.10385824 | 0.528322352 | ENSAPLG00000013997\|ENSAPLG00000001480 |
| map03010 | Ribosome | 0.08146496 | 0.529522238 | ENSAPLG00000017181\|ENSAPLG00000014350 |
| map04970 | Salivary secretion | 0.280608961 | 0.529536264 | ENSAPLG00000001480 |
| map04911 | Insulin secretion | 0.290654379 | 0.531352537 | ENSAPLG00000001480 |
| map04960 | Aldosterone-regulated sodium reabsorption | 0.118281953 | 0.532268787 | ENSAPLG00000001480 |
| map04146 | Peroxisome | 0.278075894 | 0.533358681 | MSTRG.13593 |
| map04630 | JAK-STAT signaling pathway | 0.109417729 | 0.533411431 | ENSAPLG00000004865\|ENSAPLG00000013997 |
| map04668 | TNF signaling pathway | 0.310334385 | 0.533957692 | ENSAPLG00000004865 |
| map05210 | Colorectal cancer | 0.288155994 | 0.535146845 | ENSAPLG00000013997 |
| map04964 | Proximal tubule bicarbonate reclamation | 0.096411627 | 0.537150491 | ENSAPLG00000001480 |
| map04612 | Antigen processing and presentation | 0.303017927 | 0.537168143 | MSTRG.12361 |
| map04657 | IL-17 signaling pathway | 0.307903972 | 0.537683055 | ENSAPLG00000011932 |
| map04012 | ErbB signaling pathway | 0.317575531 | 0.538497639 | ENSAPLG00000013997 |
| map04972 | Pancreatic secretion | 0.322361496 | 0.538804215 | ENSAPLG00000001480 |
| map00350 | Tyrosine metabolism | 0.115189694 | 0.53908777 | ENSAPLG00000016019 |
| map00140 | Steroid hormone biosynthesis | 0.148627603 | 0.543419675 | ENSAPLG00000018039 |
| map00620 | Pyruvate metabolism | 0.148627603 | 0.543419675 | ENSAPLG00000022939 |
| map05169 | Epstein-Barr virus infection | 0.102552974 | 0.545395363 | ENSAPLG00000007512\|ENSAPLG00000013997\|MSTRG.12361 |
| map00062 | Fatty acid elongation | 0.093244192 | 0.545478523 | ENSAPLG00000002641 |
| map04610 | Complement and coagulation cascades | 0.331834915 | 0.546826551 | ENSAPLG00000027465 |
| map05222 | Small cell lung cancer | 0.336522811 | 0.546849568 | ENSAPLG00000013997 |
| map04974 | Protein digestion and absorption | 0.336522811 | 0.546849568 | ENSAPLG00000001480 |
| map04218 | Cellular senescence | 0.1359364 | 0.548433061 | ENSAPLG00000013997\|MSTRG.12361 |
| map00830 | Retinol metabolism | 0.142641547 | 0.556302034 | ENSAPLG00000018039 |
| map05216 | Thyroid cancer | 0.142641547 | 0.556302034 | ENSAPLG00000013997 |
| map05166 | Human T-cell leukemia virus 1 infection | 0.081456478 | 0.560612229 | ENSAPLG00000004865\|ENSAPLG00000013997\|MSTRG.12361 |
| map05034 | Alcoholism | 0.35495406 | 0.561211149 | ENSAPLG00000017309 |
| map00240 | Pyrimidine metabolism | 0.03389542 | 0.566537738 | ENSAPLG00000029583\|ENSAPLG00000004498 |
| map05206 | MicroRNAs in cancer | 0.497998851 | 0.571233977 | ENSAPLG00000013997 |
| map04062 | Chemokine signaling pathway | 0.483583224 | 0.571507447 | ENSAPLG00000027336 |
| map04144 | Endocytosis | 0.078252618 | 0.572222266 | MSTRG.12361\|ENSAPLG00000004165\|ENSAPLG00000022346 |
| map04514 | Cell adhesion molecules | 0.505057971 | 0.573706627 | MSTRG.12361 |
| map05161 | Hepatitis B | 0.490841053 | 0.574284032 | ENSAPLG00000013997 |
| map05416 | Viral myocarditis | 0.451589304 | 0.574303789 | MSTRG.12361 |
| map04141 | Protein processing in endoplasmic reticulum | 0.476223995 | 0.57441451 | ENSAPLG00000023946 |
| map05330 | Allograft rejection | 0.368447155 | 0.574777561 | MSTRG.12361 |
| map04022 | cGMP-PKG signaling pathway | 0.496218705 | 0.574827609 | ENSAPLG00000001480 |
| map05150 | Staphylococcus aureus infection | 0.481752977 | 0.575154065 | ENSAPLG00000011932 |
| map04310 | Wnt signaling pathway | 0.44964796 | 0.578118806 | ENSAPLG00000013997 |
| map04390 | Hippo signaling pathway | 0.474368182 | 0.578136222 | ENSAPLG00000013997 |
| map00061 | Fatty acid biosynthesis | 0.064243047 | 0.578187419 | ENSAPLG00000002703 |
| map00120 | Primary bile acid biosynthesis | 0.064243047 | 0.578187419 | MSTRG.13593 |
| map00360 | Phenylalanine metabolism | 0.064243047 | 0.578187419 | ENSAPLG00000016019 |
| map05322 | Systemic lupus erythematosus | 0.445745038 | 0.579468549 | ENSAPLG00000017309 |
| map05323 | Rheumatoid arthritis | 0.470637195 | 0.579626862 | ENSAPLG00000004865 |
| map05226 | Gastric cancer | 0.461195766 | 0.580214029 | ENSAPLG00000013997 |
| map05224 | Breast cancer | 0.441814984 | 0.580812956 | ENSAPLG00000013997 |
| map05225 | Hepatocellular carcinoma | 0.468761978 | 0.583459058 | ENSAPLG00000013997 |
| map05231 | Choline metabolism in cancer | 0.439839725 | 0.584786906 | ENSAPLG00000017233 |
| map05160 | Hepatitis C | 0.425819734 | 0.58612834 | ENSAPLG00000013997 |
| map05165 | Human papillomavirus infection | 0.387384969 | 0.588623914 | ENSAPLG00000007512\|MSTRG.12361 |
| map04910 | Insulin signaling pathway | 0.437857613 | 0.588842997 | ENSAPLG00000002703 |
| map04976 | Bile secretion | 0.030241292 | 0.589705203 | MSTRG.13593\|ENSAPLG00000001480 |
| map04261 | Adrenergic signaling in cardiomyocytes | 0.423789 | 0.590277535 | ENSAPLG00000001480 |
| map05320 | Autoimmune thyroid disease | 0.383839611 | 0.590910979 | MSTRG.12361 |
| map04550 | Signaling pathways regulating pluripotency of stem cells | 0.415595412 | 0.592983698 | ENSAPLG00000013997 |
| map04932 | Non-alcoholic fatty liver disease | 0.415595412 | 0.592983698 | ENSAPLG00000023946 |
| map04621 | NOD-like receptor signaling pathway | 0.435868626 | 0.592984061 | ENSAPLG00000011932 |
| map04658 | Th1 and Th2 cell differentiation | 0.400980917 | 0.593857814 | ENSAPLG00000007512 |
| map04110 | Cell cycle | 0.411455893 | 0.594325179 | ENSAPLG00000013997 |
| map04152 | AMPK signaling pathway | 0.398864095 | 0.598296143 | ENSAPLG00000002703 |
| map04217 | Necroptosis | 0.409375367 | 0.598711474 | ENSAPLG00000017309 |
| map00920 | Sulfur metabolism | 0.041059709 | 0.600498251 | ENSAPLG00000022790 |
| map00430 | Taurine and hypotaurine metabolism | 0.041059709 | 0.600498251 | MSTRG.13593 |
| map05017 | Spinocerebellar ataxia | 0.062754165 | 0.611853112 | ENSAPLG00000007512\|ENSAPLG00000023946 |
| map05132 | Salmonella infection | 0.591345141 | 0.623309743 | ENSAPLG00000013997 |
| map04145 | Phagosome | 0.570506635 | 0.623825012 | MSTRG.12361 |
| map05202 | Transcriptional misregulation in cancer | 0.586964892 | 0.624317204 | ENSAPLG00000013997 |
| map04024 | cAMP signaling pathway | 0.568978935 | 0.628023919 | ENSAPLG00000001480 |
| map05168 | Herpes simplex virus 1 infection | 0.585494606 | 0.628466687 | MSTRG.12361 |
| map04714 | Thermogenesis | 0.5643638 | 0.62886252 | ENSAPLG00000021584 |
| map05205 | Proteoglycans in cancer | 0.584019189 | 0.632687455 | ENSAPLG00000013997 |
| map05170 | Human immunodeficiency virus 1 infection | 0.562814682 | 0.633166517 | MSTRG.12361 |
| map05200 | Pathways in cancer | 0.623758271 | 0.651604622 | ENSAPLG00000004865\|ENSAPLG00000013997 |
| map03008 | Ribosome biogenesis in eukaryotes | 0.662832808 | 0.686295917 | ENSAPLG00000018735 |
| map05010 | Alzheimer disease | 0.688303674 | 0.706416928 | ENSAPLG00000023946 |
| map04010 | MAPK signaling pathway | 0.698171009 | 0.710313114 | ENSAPLG00000013997 |
| map00450 | Selenocompound metabolism | 0.060965101 | 0.713291685 | ENSAPLG00000009934 |
| map00670 | One carbon pool by folate | 0.060965101 | 0.713291685 | ENSAPLG00000009934 |
| map03013 | RNA transport | 0.766827827 | 0.773438412 | MSTRG.11374 |
| map04151 | PI3K-Akt signaling pathway | 0.783009217 | 0.783009217 | ENSAPLG00000013997 |

**Supplementary Table 4.** KEGG enrichment analysis of DEGs in S1600 and C groups.

| Pathway ID | Description | P value | P adjust | Gene IDs |
| --- | --- | --- | --- | --- |
| map04145 | Phagosome | 0.008324549 | 0.26876401 | ENSAPLG00000003908\|ENSAPLG00000027345\|ENSAPLG00000030106\|ENSAPLG00000019896\|ENSAPLG00000008961\|ENSAPLG00000000588\|ENSAPLG00000012624\|ENSAPLG00000031161 |
| map04915 | Estrogen signaling pathway | 0.004908754 | 0.277344621 | ENSAPLG00000003627\|ENSAPLG00000021187\|ENSAPLG00000026143\|ENSAPLG00000030662\|MSTRG.13761\|ENSAPLG00000010667 |
| map04610 | Complement and coagulation cascades | 0.002583153 | 0.29189629 | ENSAPLG00000002995\|ENSAPLG00000028561\|MSTRG.13004\|ENSAPLG00000029863\|ENSAPLG00000001977\|ENSAPLG00000009032 |
| map01040 | Biosynthesis of unsaturated fatty acids | 0.008317813 | 0.313304296 | MSTRG.13593\|ENSAPLG00000002641\|ENSAPLG00000015665 |
| map05150 | Staphylococcus aureus infection | 0.00728882 | 0.329454644 | ENSAPLG00000002995\|MSTRG.13004\|ENSAPLG00000019896\|ENSAPLG00000011932\|ENSAPLG00000029863\|ENSAPLG00000001977\|ENSAPLG00000031161 |
| map04612 | Antigen processing and presentation | 0.001476108 | 0.33360033 | ENSAPLG00000030106\|ENSAPLG00000030662\|ENSAPLG00000026143\|ENSAPLG00000019896\|ENSAPLG00000012624\|ENSAPLG00000010667 |
| map00230 | Purine metabolism | 0.016940144 | 0.348042956 | ENSAPLG00000009439\|MSTRG.13761\|ENSAPLG00000010946\|ENSAPLG00000009106\|ENSAPLG00000029583\|ENSAPLG00000007240 |
| map05322 | Systemic lupus erythematosus | 0.015655649 | 0.353817672 | ENSAPLG00000002995\|ENSAPLG00000019896\|ENSAPLG00000029863\|ENSAPLG00000017309\|ENSAPLG00000031161\|ENSAPLG00000009032 |
| map03320 | PPAR signaling pathway | 0.004729746 | 0.356307497 | ENSAPLG00000015665\|ENSAPLG00000005745\|ENSAPLG00000001196\|ENSAPLG00000014661\|MSTRG.10972\|ENSAPLG00000026694 |
| map05020 | Prion disease | 0.01285936 | 0.363276933 | ENSAPLG00000029863\|ENSAPLG00000002995\|ENSAPLG00000009032 |
| map00270 | Cysteine and methionine metabolism | 0.023040962 | 0.371946956 | ENSAPLG00000009685\|ENSAPLG00000003607\|ENSAPLG00000005388 |
| map04146 | Peroxisome | 0.025381003 | 0.382407115 | ENSAPLG00000010946\|MSTRG.13593\|ENSAPLG00000014661\|ENSAPLG00000023048 |
| map00020 | Citrate cycle (TCA cycle) | 0.015566582 | 0.390894178 | ENSAPLG00000020285\|ENSAPLG00000016067\|MSTRG.10972 |
| map00061 | Fatty acid biosynthesis | 0.021293171 | 0.401021392 | ENSAPLG00000002703\|ENSAPLG00000014661 |
| map00120 | Primary bile acid biosynthesis | 0.021293171 | 0.401021392 | ENSAPLG00000023048\|MSTRG.13593 |
| map04217 | Necroptosis | 0.034254742 | 0.48384823 | ENSAPLG00000017309\|ENSAPLG00000030662\|ENSAPLG00000026143\|ENSAPLG00000008961\|ENSAPLG00000022295 |
| map04910 | Insulin signaling pathway | 0.047235413 | 0.533760171 | ENSAPLG00000001908\|ENSAPLG00000002703\|MSTRG.13497\|ENSAPLG00000022276\|MSTRG.10972 |
| map05217 | Basal cell carcinoma | 0.05146671 | 0.553879833 | ENSAPLG00000024305\|ENSAPLG00000009975\|ENSAPLG00000024935 |
| map04024 | cAMP signaling pathway | 0.067615075 | 0.565963219 | ENSAPLG00000024305\|ENSAPLG00000009975\|MSTRG.13761\|ENSAPLG00000011285\|ENSAPLG00000025519\|ENSAPLG00000007240 |
| map04115 | p53 signaling pathway | 0.070961632 | 0.572761745 | ENSAPLG00000003908\|ENSAPLG00000024935\|ENSAPLG00000012991 |
| map04976 | Bile secretion | 0.066828684 | 0.58089548 | MSTRG.13593\|ENSAPLG00000011285\|MSTRG.13761 |
| map05221 | Acute myeloid leukemia | 0.064805117 | 0.585838257 | ENSAPLG00000002963\|ENSAPLG00000021187\|MSTRG.12975 |
| map00220 | Arginine biosynthesis | 0.04678443 | 0.587404514 | ENSAPLG00000012495\|ENSAPLG00000015602 |
| map04964 | Proximal tubule bicarbonate reclamation | 0.04678443 | 0.587404514 | ENSAPLG00000011285\|MSTRG.10972 |
| map04923 | Regulation of lipolysis in adipocytes | 0.06084521 | 0.597870321 | ENSAPLG00000014313\|ENSAPLG00000007890\|MSTRG.13761 |
| map00983 | Drug metabolism - other enzymes | 0.06084521 | 0.597870321 | ENSAPLG00000010946\|ENSAPLG00000029583\|ENSAPLG00000004498 |
| map00232 | Caffeine metabolism | 0.058410386 | 0.600033965 | ENSAPLG00000010946 |
| map04550 | Signaling pathways regulating pluripotency of stem cells | 0.111463364 | 0.614407812 | ENSAPLG00000020045\|MSTRG.3343\|MSTRG.13671\|ENSAPLG00000026069 |
| map04621 | NOD-like receptor signaling pathway | 0.046223672 | 0.614502939 | ENSAPLG00000008961\|ENSAPLG00000015226\|ENSAPLG00000030662\|ENSAPLG00000026143\|ENSAPLG00000011932 |
| map04210 | Apoptosis | 0.117410043 | 0.617085343 | ENSAPLG00000012624\|ENSAPLG00000012991\|ENSAPLG00000022295\|ENSAPLG00000024935 |
| map05140 | Leishmaniasis | 0.109511984 | 0.61874271 | ENSAPLG00000008961\|ENSAPLG00000031161\|ENSAPLG00000019896\|ENSAPLG00000012199 |
| map04350 | TGF-beta signaling pathway | 0.115478012 | 0.621381684 | ENSAPLG00000026069\|ENSAPLG00000003908\|ENSAPLG00000012199 |
| map05414 | Dilated cardiomyopathy | 0.151324973 | 0.621808072 | ENSAPLG00000027827\|ENSAPLG00000031161\|MSTRG.13761\|ENSAPLG00000012199 |
| map05219 | Bladder cancer | 0.148650578 | 0.622130196 | ENSAPLG00000002776\|ENSAPLG00000003908 |
| map00590 | Arachidonic acid metabolism | 0.140401554 | 0.62217159 | ENSAPLG00000008235\|ENSAPLG00000014313 |
| map00072 | Synthesis and degradation of ketone bodies | 0.124040998 | 0.622961458 | ENSAPLG00000005372 |
| map05200 | Pathways in cancer | 0.104907095 | 0.623921141 | ENSAPLG00000002776\|ENSAPLG00000021187\|MSTRG.12975\|ENSAPLG00000002963\|MSTRG.13761\|ENSAPLG00000030662\|ENSAPLG00000026143\|ENSAPLG00000024305\|ENSAPLG00000024935\|ENSAPLG00000009975\|ENSAPLG00000012199 |
| map00603 | Glycosphingolipid biosynthesis - globo and isoglobo series | 0.155129822 | 0.626059638 | ENSAPLG00000019280 |
| map00830 | Retinol metabolism | 0.097186063 | 0.627544293 | ENSAPLG00000008235\|ENSAPLG00000018151 |
| map04961 | Endocrine and other factor-regulated calcium reabsorption | 0.144513537 | 0.628078064 | ENSAPLG00000011285\|MSTRG.13761 |
| map00260 | Glycine, serine and threonine metabolism | 0.108557484 | 0.629076702 | ENSAPLG00000009685\|ENSAPLG00000005388 |
| map04972 | Pancreatic secretion | 0.147804211 | 0.630259466 | ENSAPLG00000011285\|ENSAPLG00000023455\|MSTRG.13761 |
| map04514 | Cell adhesion molecules | 0.092353455 | 0.632481235 | ENSAPLG00000027519\|ENSAPLG00000015814\|ENSAPLG00000019896\|ENSAPLG00000030106\|ENSAPLG00000000603 |
| map05202 | Transcriptional misregulation in cancer | 0.081236777 | 0.633086606 | ENSAPLG00000021187\|MSTRG.12975\|ENSAPLG00000002963\|ENSAPLG00000007495\|ENSAPLG00000024935\|ENSAPLG00000031161 |
| map00430 | Taurine and hypotaurine metabolism | 0.134528296 | 0.633404058 | MSTRG.13593 |
| map05146 | Amoebiasis | 0.123492628 | 0.634303041 | ENSAPLG00000015602\|ENSAPLG00000031161\|ENSAPLG00000012199\|ENSAPLG00000009928 |
| map00330 | Arginine and proline metabolism | 0.16120039 | 0.639145405 | ENSAPLG00000007914\|ENSAPLG00000015602 |
| map04068 | FoxO signaling pathway | 0.101864256 | 0.639481162 | ENSAPLG00000024935\|ENSAPLG00000012199\|ENSAPLG00000022295\|MSTRG.10972 |
| map00620 | Pyruvate metabolism | 0.104728251 | 0.639691476 | ENSAPLG00000025865\|MSTRG.10972 |
| map04152 | AMPK signaling pathway | 0.096297733 | 0.640096695 | ENSAPLG00000002703\|ENSAPLG00000015665\|MSTRG.13497\|MSTRG.10972 |
| map04657 | IL-17 signaling pathway | 0.131286043 | 0.645014037 | ENSAPLG00000011932\|ENSAPLG00000030662\|ENSAPLG00000026143 |
| map04914 | Progesterone-mediated oocyte maturation | 0.131286043 | 0.645014037 | ENSAPLG00000026143\|ENSAPLG00000030662\|MSTRG.13761 |
| map04640 | Hematopoietic cell lineage | 0.140347702 | 0.647317974 | MSTRG.7165\|ENSAPLG00000024808\|ENSAPLG00000031161\|ENSAPLG00000019896 |
| map04659 | Th17 cell differentiation | 0.140347702 | 0.647317974 | ENSAPLG00000021187\|ENSAPLG00000030662\|ENSAPLG00000026143\|ENSAPLG00000019896 |
| map00750 | Vitamin B6 metabolism | 0.09181497 | 0.648443224 | ENSAPLG00000005388 |
| map05142 | Chagas disease | 0.170800095 | 0.665531404 | ENSAPLG00000029863\|ENSAPLG00000002995\|ENSAPLG00000012199 |
| map04931 | Insulin resistance | 0.182663496 | 0.665837905 | ENSAPLG00000001908\|MSTRG.13497\|MSTRG.10972 |
| map04061 | Viral protein interaction with cytokine and cytokine receptor | 0.186825021 | 0.670197695 | ENSAPLG00000027336\|ENSAPLG00000022295 |
| map04340 | Hedgehog signaling pathway | 0.186825021 | 0.670197695 | ENSAPLG00000024305\|ENSAPLG00000009975 |
| map04213 | Longevity regulating pathway - multiple species | 0.182512636 | 0.676194355 | ENSAPLG00000010667\|MSTRG.13761 |
| map00071 | Fatty acid degradation | 0.08980889 | 0.676560307 | ENSAPLG00000014661\|ENSAPLG00000026694 |
| map04216 | Ferroptosis | 0.08980889 | 0.676560307 | ENSAPLG00000008961\|ENSAPLG00000014661 |
| map04512 | ECM-receptor interaction | 0.179676731 | 0.676782354 | ENSAPLG00000003908\|ENSAPLG00000024808\|ENSAPLG00000027519 |
| map00670 | One carbon pool by folate | 0.194881376 | 0.677587552 | ENSAPLG00000009771 |
| map04141 | Protein processing in endoplasmic reticulum | 0.178926163 | 0.685378184 | ENSAPLG00000005104\|ENSAPLG00000010667\|ENSAPLG00000030662\|ENSAPLG00000026143 |
| map04918 | Thyroid hormone synthesis | 0.208583062 | 0.693231942 | ENSAPLG00000011285\|MSTRG.13761 |
| map05330 | Allograft rejection | 0.207010052 | 0.698272712 | ENSAPLG00000031161\|ENSAPLG00000019896\|ENSAPLG00000030106 |
| map04013 | MAPK signaling pathway - fly | 0.292670172 | 0.703653819 | ENSAPLG00000017871\|ENSAPLG00000028616 |
| map04022 | cGMP-PKG signaling pathway | 0.205590727 | 0.703992488 | ENSAPLG00000025519\|ENSAPLG00000011285\|MSTRG.13761\|ENSAPLG00000003320 |
| map05133 | Pertussis | 0.230586126 | 0.704222493 | ENSAPLG00000029863\|ENSAPLG00000002995 |
| map04261 | Adrenergic signaling in cardiomyocytes | 0.290010135 | 0.704755811 | ENSAPLG00000011285\|MSTRG.13761\|ENSAPLG00000013843 |
| map05145 | Toxoplasmosis | 0.296537584 | 0.705447304 | ENSAPLG00000010667\|ENSAPLG00000019896\|ENSAPLG00000012199 |
| map05166 | Human T-cell leukemia virus 1 infection | 0.271708226 | 0.70581677 | ENSAPLG00000030106\|ENSAPLG00000003320\|MSTRG.13761\|ENSAPLG00000019896\|ENSAPLG00000012199 |
| map04714 | Thermogenesis | 0.312671578 | 0.706637767 | MSTRG.13761\|ENSAPLG00000014661\|ENSAPLG00000021584\|ENSAPLG00000026694 |
| map04920 | Adipocytokine signaling pathway | 0.235006953 | 0.708154285 | ENSAPLG00000014661\|MSTRG.10972 |
| map04270 | Vascular smooth muscle contraction | 0.238383717 | 0.708877895 | ENSAPLG00000025519\|ENSAPLG00000002915\|MSTRG.13761 |
| map05418 | Fluid shear stress and atherosclerosis | 0.270504114 | 0.710859648 | ENSAPLG00000027519\|ENSAPLG00000030662\|ENSAPLG00000026143 |
| map00512 | Mucin type O-glycan biosynthesis | 0.303317434 | 0.714059791 | ENSAPLG00000015618 |
| map04611 | Platelet activation | 0.244757924 | 0.7183804 | ENSAPLG00000014313\|ENSAPLG00000024808\|MSTRG.13761 |
| map04672 | Intestinal immune network for IgA production | 0.228879048 | 0.7184259 | ENSAPLG00000031161\|ENSAPLG00000019896\|ENSAPLG00000027336 |
| map05320 | Autoimmune thyroid disease | 0.228879048 | 0.7184259 | ENSAPLG00000031161\|ENSAPLG00000019896\|ENSAPLG00000030106 |
| map04950 | Maturity onset diabetes of the young | 0.311669225 | 0.718747396 | ENSAPLG00000020045 |
| map00591 | Linoleic acid metabolism | 0.311669225 | 0.718747396 | ENSAPLG00000008235 |
| map00062 | Fatty acid elongation | 0.286310394 | 0.718957211 | ENSAPLG00000002641 |
| map00601 | Glycosphingolipid biosynthesis - lacto and neolacto series | 0.286310394 | 0.718957211 | ENSAPLG00000019280 |
| map01523 | Antifolate resistance | 0.286310394 | 0.718957211 | ENSAPLG00000009771 |
| map05144 | Malaria | 0.270490058 | 0.71918533 | ENSAPLG00000003908\|ENSAPLG00000012199 |
| map05332 | Graft-versus-host disease | 0.226170979 | 0.719924524 | ENSAPLG00000019896\|ENSAPLG00000030106 |
| map04060 | Cytokine-cytokine receptor interaction | 0.280456122 | 0.720262313 | ENSAPLG00000026069\|ENSAPLG00000012199\|ENSAPLG00000022295\|ENSAPLG00000027336 |
| map05220 | Chronic myeloid leukemia | 0.28380472 | 0.720672661 | ENSAPLG00000024935\|ENSAPLG00000012199 |
| map04970 | Salivary secretion | 0.310354383 | 0.723093716 | ENSAPLG00000011285\|MSTRG.13761 |
| map04010 | MAPK signaling pathway | 0.220910909 | 0.723563267 | ENSAPLG00000009896\|ENSAPLG00000003320\|ENSAPLG00000024935\|ENSAPLG00000022276\|ENSAPLG00000010667\|ENSAPLG00000012199 |
| map05210 | Colorectal cancer | 0.323560179 | 0.724005945 | ENSAPLG00000024935\|ENSAPLG00000012199 |
| map04940 | Type I diabetes mellitus | 0.26604984 | 0.724424866 | ENSAPLG00000019896\|ENSAPLG00000030106 |
| map04971 | Gastric acid secretion | 0.26604984 | 0.724424866 | ENSAPLG00000011285\|MSTRG.13761 |
| map05321 | Inflammatory bowel disease | 0.336702241 | 0.72471149 | ENSAPLG00000019896\|ENSAPLG00000012199 |
| map04911 | Insulin secretion | 0.327948562 | 0.726631128 | ENSAPLG00000011285\|MSTRG.13761 |
| map00900 | Terpenoid backbone biosynthesis | 0.251048147 | 0.727395913 | ENSAPLG00000031181 |
| map04142 | Lysosome | 0.26403492 | 0.727705998 | ENSAPLG00000012624\|ENSAPLG00000026479\|ENSAPLG00000020610 |
| map04670 | Leukocyte transendothelial migration | 0.225727179 | 0.728776322 | ENSAPLG00000008961\|ENSAPLG00000025519\|ENSAPLG00000009928 |
| map04064 | NF-kappa B signaling pathway | 0.332547268 | 0.729666822 | ENSAPLG00000031161\|ENSAPLG00000024935\|ENSAPLG00000012991 |
| map05416 | Viral myocarditis | 0.33582305 | 0.72976932 | ENSAPLG00000031161\|ENSAPLG00000019896\|ENSAPLG00000030106 |
| map05310 | Asthma | 0.371347437 | 0.729778441 | ENSAPLG00000031161\|ENSAPLG00000019896 |
| map04933 | AGE-RAGE signaling pathway in diabetic complications | 0.371347437 | 0.729778441 | ENSAPLG00000008961\|ENSAPLG00000012199 |
| map04975 | Fat digestion and absorption | 0.397221107 | 0.729853416 | ENSAPLG00000001196 |
| map05152 | Tuberculosis | 0.39411621 | 0.730084128 | ENSAPLG00000012624\|ENSAPLG00000031161\|ENSAPLG00000019896\|ENSAPLG00000012199 |
| map05323 | Rheumatoid arthritis | 0.368507467 | 0.73054989 | ENSAPLG00000031161\|ENSAPLG00000019896\|ENSAPLG00000012199 |
| map04218 | Cellular senescence | 0.38151402 | 0.730696343 | ENSAPLG00000030106\|ENSAPLG00000024935\|ENSAPLG00000012199 |
| map04724 | Glutamatergic synapse | 0.417754011 | 0.731879119 | MSTRG.13761\|ENSAPLG00000017196 |
| map05216 | Thyroid cancer | 0.411598877 | 0.732451545 | ENSAPLG00000024935 |
| map05169 | Epstein-Barr virus infection | 0.344150324 | 0.733754465 | ENSAPLG00000007512\|ENSAPLG00000031161\|ENSAPLG00000019896\|ENSAPLG00000030106\|ENSAPLG00000024935 |
| map04727 | GABAergic synapse | 0.379900951 | 0.733825768 | MSTRG.13761\|ENSAPLG00000017196 |
| map04540 | Gap junction | 0.354105159 | 0.734199688 | ENSAPLG00000027345\|MSTRG.13761 |
| map00650 | Butanoate metabolism | 0.367409325 | 0.734818649 | ENSAPLG00000005372 |
| map05132 | Salmonella infection | 0.361533301 | 0.73609483 | ENSAPLG00000025519\|ENSAPLG00000030662\|ENSAPLG00000026143\|ENSAPLG00000022295 |
| map05206 | MicroRNAs in cancer | 0.416949763 | 0.736176924 | ENSAPLG00000003908\|ENSAPLG00000003607\|ENSAPLG00000023484 |
| map04960 | Aldosterone-regulated sodium reabsorption | 0.351958241 | 0.736505208 | ENSAPLG00000011285 |
| map04726 | Serotonergic synapse | 0.388405765 | 0.737644563 | ENSAPLG00000008235\|ENSAPLG00000014313 |
| map05410 | Hypertrophic cardiomyopathy | 0.405259357 | 0.738617861 | ENSAPLG00000027827\|ENSAPLG00000012199 |
| map05032 | Morphine addiction | 0.454417874 | 0.738837695 | ENSAPLG00000007240\|MSTRG.13761 |
| map00240 | Pyrimidine metabolism | 0.261609667 | 0.739047308 | ENSAPLG00000029583\|ENSAPLG00000004498 |
| map05212 | Pancreatic cancer | 0.261609667 | 0.739047308 | ENSAPLG00000024935\|ENSAPLG00000012199 |
| map03030 | DNA replication | 0.359730123 | 0.739081889 | ENSAPLG00000015834 |
| map04925 | Aldosterone synthesis and secretion | 0.392639117 | 0.739470337 | ENSAPLG00000011285\|MSTRG.13761 |
| map04922 | Glucagon signaling pathway | 0.392639117 | 0.739470337 | ENSAPLG00000004813\|MSTRG.10972 |
| map00140 | Steroid hormone biosynthesis | 0.425635615 | 0.739951145 | ENSAPLG00000008235 |
| map04974 | Protein digestion and absorption | 0.409438554 | 0.740264906 | ENSAPLG00000011285\|ENSAPLG00000017196 |
| map04750 | Inflammatory mediator regulation of TRP channels | 0.409438554 | 0.740264906 | ENSAPLG00000008235\|MSTRG.13761 |
| map04510 | Focal adhesion | 0.350654006 | 0.740633694 | ENSAPLG00000003908\|ENSAPLG00000025519\|ENSAPLG00000003801\|ENSAPLG00000009928 |
| map05215 | Prostate cancer | 0.367053247 | 0.740661016 | ENSAPLG00000030662\|ENSAPLG00000026143 |
| map05203 | Viral carcinogenesis | 0.429681288 | 0.741282222 | ENSAPLG00000007512\|ENSAPLG00000030106\|ENSAPLG00000003320 |
| map05204 | Chemical carcinogenesis | 0.452717979 | 0.741407705 | ENSAPLG00000008235 |
| map04928 | Parathyroid hormone synthesis, secretion and action | 0.434206226 | 0.743413689 | ENSAPLG00000007240\|MSTRG.13761 |
| map00564 | Glycerophospholipid metabolism | 0.434206226 | 0.743413689 | ENSAPLG00000008156\|ENSAPLG00000013386 |
| map00100 | Steroid biosynthesis | 0.260022851 | 0.743862838 | ENSAPLG00000020771 |
| map04066 | HIF-1 signaling pathway | 0.442339295 | 0.74603493 | ENSAPLG00000022276\|ENSAPLG00000008961 |
| map00380 | Tryptophan metabolism | 0.44606883 | 0.746752263 | ENSAPLG00000013859 |
| map04978 | Mineral absorption | 0.44606883 | 0.746752263 | ENSAPLG00000011285 |
| map04973 | Carbohydrate digestion and absorption | 0.44606883 | 0.746752263 | ENSAPLG00000011285 |
| map04962 | Vasopressin-regulated water reabsorption | 0.503131848 | 0.778820532 | MSTRG.13761 |
| map04080 | Neuroactive ligand-receptor interaction | 0.490046606 | 0.779933331 | MSTRG.3968\|ENSAPLG00000006947\|MSTRG.3967\|ENSAPLG00000027479\|ENSAPLG00000001977 |
| map04650 | Natural killer cell mediated cytotoxicity | 0.501191072 | 0.781166775 | ENSAPLG00000031161\|ENSAPLG00000022295 |
| map04926 | Relaxin signaling pathway | 0.485882986 | 0.784353963 | ENSAPLG00000002915\|MSTRG.13761 |
| map03010 | Ribosome | 0.485882986 | 0.784353963 | ENSAPLG00000017181\|ENSAPLG00000014350 |
| map00250 | Alanine, aspartate and glutamate metabolism | 0.497091842 | 0.78561368 | ENSAPLG00000009106 |
| map00600 | Sphingolipid metabolism | 0.497091842 | 0.78561368 | ENSAPLG00000020610 |
| map04913 | Ovarian steroidogenesis | 0.514996321 | 0.791763051 | MSTRG.13761 |
| map03040 | Spliceosome | 0.527269657 | 0.794419617 | ENSAPLG00000014681\|ENSAPLG00000010667 |
| map04330 | Notch signaling pathway | 0.53226688 | 0.796637847 | ENSAPLG00000007512 |
| map00280 | Valine, leucine and isoleucine degradation | 0.526579068 | 0.798703822 | ENSAPLG00000021074 |
| map04658 | Th1 and Th2 cell differentiation | 0.523600491 | 0.799552101 | ENSAPLG00000007512\|ENSAPLG00000019896 |
| map05134 | Legionellosis | 0.537886742 | 0.799752656 | ENSAPLG00000010667 |
| map04110 | Cell cycle | 0.541755585 | 0.800240276 | ENSAPLG00000024935\|ENSAPLG00000012199 |
| map04371 | Apelin signaling pathway | 0.559429192 | 0.820980502 | ENSAPLG00000002915\|MSTRG.13761 |
| map04742 | Taste transduction | 0.580494355 | 0.825105184 | MSTRG.13761 |
| map05168 | Herpes simplex virus 1 infection | 0.578003307 | 0.826764224 | ENSAPLG00000014681\|ENSAPLG00000019896\|ENSAPLG00000030106 |
| map00010 | Glycolysis / Gluconeogenesis | 0.575389816 | 0.828268143 | MSTRG.10972 |
| map05213 | Endometrial cancer | 0.570223524 | 0.831422686 | ENSAPLG00000024935 |
| map05205 | Proteoglycans in cancer | 0.575275115 | 0.833411385 | ENSAPLG00000003908\|ENSAPLG00000027519\|ENSAPLG00000023484 |
| map04151 | PI3K-Akt signaling pathway | 0.59058321 | 0.834198784 | ENSAPLG00000026143\|ENSAPLG00000003908\|ENSAPLG00000030662\|ENSAPLG00000031161\|MSTRG.10972 |
| map04927 | Cortisol synthesis and secretion | 0.600309535 | 0.842670528 | MSTRG.13761 |
| map05218 | Melanoma | 0.605116148 | 0.84417438 | ENSAPLG00000024935 |
| map05225 | Hepatocellular carcinoma | 0.637437435 | 0.847416825 | ENSAPLG00000024935\|ENSAPLG00000012199 |
| map00310 | Lysine degradation | 0.645869527 | 0.848642518 | MSTRG.13599 |
| map05143 | African trypanosomiasis | 0.645869527 | 0.848642518 | ENSAPLG00000031161 |
| map04921 | Oxytocin signaling pathway | 0.643427555 | 0.850377938 | ENSAPLG00000025519\|MSTRG.13761 |
| map05214 | Glioma | 0.614557626 | 0.852086034 | ENSAPLG00000024935 |
| map04924 | Renin secretion | 0.637190788 | 0.85210129 | MSTRG.13761 |
| map04212 | Longevity regulating pathway - worm | 0.619193849 | 0.853279329 | ENSAPLG00000015665 |
| map04062 | Chemokine signaling pathway | 0.660932195 | 0.853546721 | MSTRG.13761\|ENSAPLG00000027336 |
| map05130 | Pathogenic Escherichia coli infection | 0.635198827 | 0.85449366 | ENSAPLG00000027345\|ENSAPLG00000031161\|ENSAPLG00000022295 |
| map04260 | Cardiac muscle contraction | 0.65850208 | 0.855295805 | ENSAPLG00000011285 |
| map05223 | Non-small cell lung cancer | 0.628300603 | 0.855397206 | ENSAPLG00000024935 |
| map05211 | Renal cell carcinoma | 0.628300603 | 0.855397206 | ENSAPLG00000012199 |
| map05226 | Gastric cancer | 0.625222738 | 0.85636569 | ENSAPLG00000024935\|ENSAPLG00000012199 |
| map05340 | Primary immunodeficiency | 0.674651118 | 0.866313368 | ENSAPLG00000031161 |
| map04211 | Longevity regulating pathway | 0.678568287 | 0.866420525 | MSTRG.13761 |
| map04520 | Adherens junction | 0.68243856 | 0.866466936 | ENSAPLG00000009928 |
| map05100 | Bacterial invasion of epithelial cells | 0.693773553 | 0.875937559 | ENSAPLG00000009928 |
| map04935 | Growth hormone synthesis, secretion and action | 0.77928626 | 0.880593474 | MSTRG.13761 |
| map05034 | Alcoholism | 0.77928626 | 0.880593474 | ENSAPLG00000017309 |
| map04916 | Melanogenesis | 0.711778109 | 0.883856333 | MSTRG.13761 |
| map05164 | Influenza A | 0.709341833 | 0.885697538 | ENSAPLG00000019896\|ENSAPLG00000022295 |
| map04810 | Regulation of actin cytoskeleton | 0.777178091 | 0.887082063 | ENSAPLG00000025519\|ENSAPLG00000009928 |
| map05163 | Human cytomegalovirus infection | 0.777178091 | 0.887082063 | MSTRG.13761\|ENSAPLG00000030106 |
| map05412 | Arrhythmogenic right ventricular cardiomyopathy | 0.708263453 | 0.889264113 | ENSAPLG00000027827 |
| map04662 | B cell receptor signaling pathway | 0.776590684 | 0.90004869 | ENSAPLG00000031161 |
| map04664 | Fc epsilon RI signaling pathway | 0.776590684 | 0.90004869 | ENSAPLG00000031161 |
| map04114 | Oocyte meiosis | 0.776590684 | 0.90004869 | MSTRG.13761 |
| map04070 | Phosphatidylinositol signaling system | 0.804525477 | 0.900112662 | ENSAPLG00000029075 |
| map05131 | Shigellosis | 0.814374204 | 0.902198873 | ENSAPLG00000025519\|ENSAPLG00000009928 |
| map04713 | Circadian entrainment | 0.762613872 | 0.90235987 | MSTRG.13761 |
| map04015 | Rap1 signaling pathway | 0.810918944 | 0.90279646 | ENSAPLG00000003908\|MSTRG.13761 |
| map04072 | Phospholipase D signaling pathway | 0.775158282 | 0.903019442 | MSTRG.13761\|ENSAPLG00000031161 |
| map05010 | Alzheimer disease | 0.759251108 | 0.903109213 | ENSAPLG00000027345\|ENSAPLG00000008961\|ENSAPLG00000021074 |
| map01522 | Endocrine resistance | 0.731999223 | 0.903999041 | MSTRG.13761 |
| map04361 | Axon regeneration | 0.738421027 | 0.906973653 | ENSAPLG00000009896 |
| map05224 | Breast cancer | 0.865919489 | 0.910222347 | ENSAPLG00000024935 |
| map04360 | Axon guidance | 0.745175755 | 0.910322814 | ENSAPLG00000025519\|ENSAPLG00000024016 |
| map00561 | Glycerolipid metabolism | 0.773862378 | 0.910900507 | ENSAPLG00000013386 |
| map04380 | Osteoclast differentiation | 0.773862378 | 0.910900507 | ENSAPLG00000013513 |
| map04725 | Cholinergic synapse | 0.7508092 | 0.912273543 | MSTRG.13761 |
| map05231 | Choline metabolism in cancer | 0.864277265 | 0.912741411 | ENSAPLG00000017233 |
| map04140 | Autophagy - animal | 0.844838228 | 0.913557127 | ENSAPLG00000002776 |
| map04020 | Calcium signaling pathway | 0.853049251 | 0.913692563 | ENSAPLG00000006947\|ENSAPLG00000031161 |
| map04144 | Endocytosis | 0.861174461 | 0.913734405 | ENSAPLG00000010667\|ENSAPLG00000030106 |
| map05222 | Small cell lung cancer | 0.756782734 | 0.914614428 | ENSAPLG00000024935 |
| map05017 | Spinocerebellar ataxia | 0.756782734 | 0.914614428 | ENSAPLG00000007512 |
| map04912 | GnRH signaling pathway | 0.756782734 | 0.914614428 | MSTRG.13761 |
| map04934 | Cushing syndrome | 0.878365478 | 0.914795382 | MSTRG.13761 |
| map04919 | Thyroid hormone signaling pathway | 0.850398853 | 0.915191146 | ENSAPLG00000011285 |
| map04150 | mTOR signaling pathway | 0.875366078 | 0.915892285 | ENSAPLG00000013386 |
| map04666 | Fc gamma R-mediated phagocytosis | 0.859229638 | 0.915971217 | ENSAPLG00000031161 |
| map04120 | Ubiquitin mediated proteolysis | 0.884150646 | 0.916596541 | ENSAPLG00000010455 |
| map05162 | Measles | 0.833093045 | 0.918434283 | ENSAPLG00000010667 |
| map04390 | Hippo signaling pathway | 0.8909988 | 0.919478213 | ENSAPLG00000012199 |
| map04723 | Retrograde endocannabinoid signaling | 0.842939395 | 0.920310644 | MSTRG.13761 |
| map04932 | Non-alcoholic fatty liver disease | 0.842939395 | 0.920310644 | MSTRG.13497 |
| map05165 | Human papillomavirus infection | 0.840745686 | 0.922371481 | ENSAPLG00000003908\|ENSAPLG00000007512\|ENSAPLG00000030106 |
| map05167 | Kaposi sarcoma-associated herpesvirus infection | 0.91032119 | 0.926723374 | ENSAPLG00000030106 |
| map05161 | Hepatitis B | 0.902326704 | 0.926935614 | ENSAPLG00000012199 |
| map04530 | Tight junction | 0.908105888 | 0.92865127 | ENSAPLG00000025519 |
| map05170 | Human immunodeficiency virus 1 infection | 0.942233587 | 0.954909375 | ENSAPLG00000030106 |
| map05016 | Huntington disease | 0.96417387 | 0.972782565 | ENSAPLG00000027345 |
| map03008 | Ribosome biogenesis in eukaryotes | 0.976402796 | 0.980742364 | ENSAPLG00000019495 |
| map03013 | RNA transport | 0.993380073 | 0.993380073 | ENSAPLG00000019495 |
